# Nano-SiO_2_/DBN: an efficacious and reusable catalyst for one-pot synthesis of tetrahydrobenzo[*b*]pyran derivatives

**DOI:** 10.1186/s13065-021-00760-3

**Published:** 2021-05-21

**Authors:** Maryam Mehravar, Bi Bi Fatemeh Mirjalili, Elaheh Babaei, Abdolhamid Bamoniri

**Affiliations:** 1 Department of Chemistry, College of Science, Yazd University, Yazd, Iran; 2 Department of Organic Chemistry, Faculty of Chemistry, University of Kashan, Kashan, Iran

**Keywords:** Nano-SiO_2_/DBN, Benzopyrans, Heterogeneous catalyst, Multicomponent reaction, Nano-silica

## Abstract

**Background:**

The nano-sized particles enhance the exposed surface area of the active part of the catalyst, thereby increasing the contact between precursors and catalyst considerably. In this study, nano-SiO_2_/1,5-diazabicyclo[4.3.0]non-5-en was synthesized, characterized and used as a heterogeneous nanocatalyst for the synthesis of tetrahydrobenzo[*b*]pyran derivatives. Fourier Transform Infrared Spectroscopy, Field Emission Scanning Electron Microscopy, Brunauer–Emmett–Teller plot, Energy Dispersive X-ray Spectroscopy and Thermo Gravimetric Analysis were used to discern nano-SiO_2_/1,5-diazabicyclo[4.3.0]non-5-en.

**Results:**

Tetrahydrobenzo[*b*]pyrans were synthesized by using nano-SiO_2_/1,5-diazabicyclo[4.3.0]non-5-en via one-pot three-component condensation of malononitrile, aldehydes and dimedone in H_2_O/EtOH at 60 °C. The results indicate that tetrahydrobenzo[*b*]pyrans were synthesized in good to high yields and short reaction times.

**Conclusions:**

The fundamental privileges of this method are short reaction time, plain procedure, recyclability of catalyst and high yields of products.

**Supplementary Information:**

The online version contains supplementary material available at 10.1186/s13065-021-00760-3.

## Introduction

Multi-component reactions (MCRs) have significant role in organic chemistry, because of some merits like selectivity, synthetic convergence, high atom economy, simplicity, short reaction time, facility of workup, synthetic efficiency and high yield of products [1, 2]. An efficient way for the synthesis of heterocyclic compounds is using multi-component reactions, which have great value in design of biologically new active compounds [1, 3–5].

**Fig. 1:**
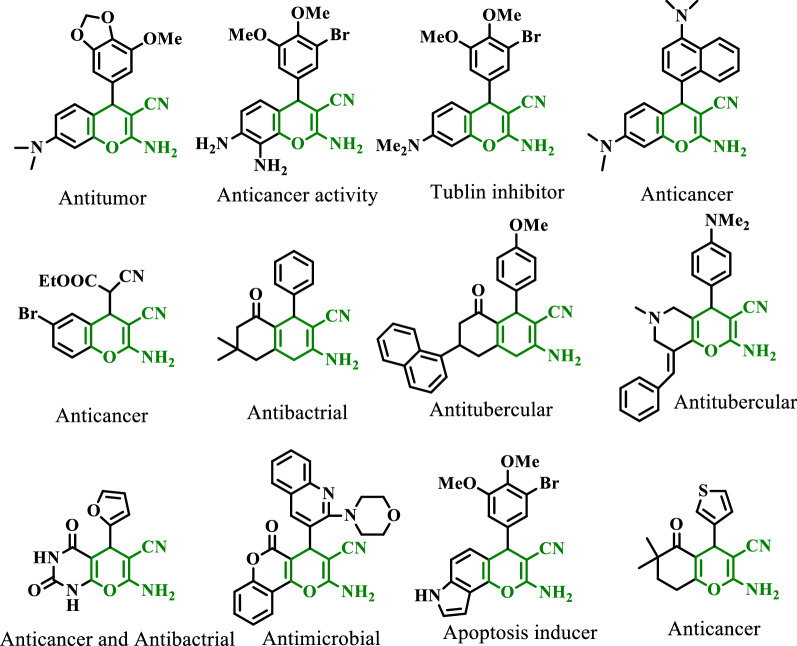
Selected examples of pharmacologically and biologically active benzopyrans

Tetrahydrobenzo[*b*]pyrans as one of the significant group of oxygen-containing heterocycle compounds are highly considered due to their medicinal and biological properties such as spasmolytic [6], antitumor [7], antibacterial [8], anti HIV [9], insulin-sensitizing activity [10] and hypotensive antiviral [11]. Some pharmacologically and biologically active benzopyrans are shown in Fig. 1.

A suitable method for synthesis of benzopyrans is three-component condensation of malononitrile and dimedone with various aldehydes. This reaction has been investigated in the presence of various catalysts such a Fe_3_O_4_@SiO_2_@NiSB [12], oxyammonium-based ionic liquid [13], WEMFSA [14], [Bmim]Sac [15], MNPs–PhSO_3_H [16], NH_2_@SiO_2_@Fe_3_O_4_ MNPs [17], choline chloride-oxalic acid [18], SCMNPs@PC/VB1-Zn [19], MMWCNTs-D-(CH_2_)4-SO_3_H [20] and Chlorophyll [21]. In this research a practical, simple and inexpensive procedure for the synthesis of tetrahydrobenzo[*b*]pyran derivatives is reported by the reaction of aldehydes, malononitrile and dimedone in the presence of catalytic amount of nano-silica supported 1,5-diazabicyclo[4.3.0]non-5-en (Nano-SiO_2_/DBN). Moreover, the amount of catalyst used in the reaction and its effect on the product yields, as well as the ability to recovery have been studied. Inexpensive and readily available catalyst, easy work-up and high yield of the products, usage of environmentally benign solvents, short reaction times and simplicity of experimental procedure are some advantages of this procedure.

## Experimental

### Materials and methods

#### General

Whole reagents and solvent were procured from Merck, Aldrich and fluka chemical companies. Fourier transform infrared spectroscopy (FT-IR) (ATR or KBr pellets) was run on a Bruker, Eqinox 55 spectrometer. The nanoparticles size and catalyst morphology were ascertained at Field emission scanning electron microscope (FE-SEM) using a Mira 3-XMU. Proton nuclear magnetic resonance (^1^H NMR) and carbon nuclear magnetic resonance (^13^C NMR) spectra were record at Bruker (DRX-400 Avance) in DMSO-d_6_ as the solvent. The crystallographic characteristics of the sample were obtained by X-ray diffractometer (XRD, Philips Xpert) using Ni-filtered CuKα (kCuK = 0.1542 nm, radiation at 40 kV and 30 Ma) in the 2θ range from 10° to 80°. Thermo gravimetric analysis (TGA) was accomplished using a STA 505 instrument under argon atomosphere. The BET surface area, pore size and pore volume were measured by using Tristar II 3020 analyzer. Melting points were recorded on a Buchi B-540 B. V. CHI apparatus. Energy Dispersive X-ray Spectroscopy (EDS) was measured by Phenom pro X.

#### Chemistry

##### General procedure for synthesis of tetrahydrobenzo[*b*]pyran derivatives

Nano-SiO_2_/DBN (0.03 g) as a nanocatalyst was combined with a mixture of dimedone (1 mmol), aromatic and aliphatic aldehyde (1 mmol), malononitrile (1 mmol) in a round bottom flask and then the mixture was stirred magnetically in H_2_O/EtOH (1:1) at 60 °C. The advancement of the reaction was controlled by TLC (*n*-hexane–ethyl acetate, 3:1). When the reaction was over, the catalyst was separated and recovered for the next run. Then, the crude products were recrystallized in EtOH.

##### Procedure for synthesis of silica chloride

Thionyl chloride (40 mL) (toxic and should be used under ventilator) and nano-silicagel (10 g) were added to a round bottomed flask (250 mL) provided with a condenser under reflux condition for 48 h. Then it was cooled to room temperature, the mixture of reaction was filtrated via a Buchner funnel, then the remainder was rinsed several times with dichloromethane. Finally, obtained sillica chloride was dried at ambient temperature.

##### Procedure for synthesis of nano-SiO_2_/DBN

Silica chloride (1 g), DBN (1.5 mL) and *n*-hexane (10 mL) were added to a round bottomed flask (100 mL) furnished with a condenser, under reflux conditions for 15 h. When reaction was completed, it was cooled, filtrated and rinsed three times with *n*-hexane. Finally, the nano-SiO_2_/DBN catalyst was dehydrated at ambient temperature in open air.

## Results and discussion

**Scheme 1:**
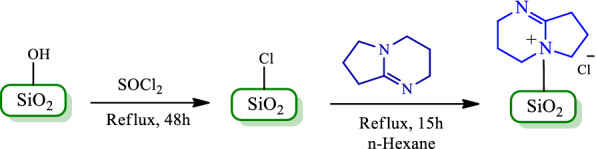
The preparation protocol for nano-SiO_2_/DBN

A new catalyst was prepared as nano-SiO_2_/DBN in two steps. At first, a mixture of thionyl chloride and commercial nano-silica gel was stirred for 48 h under reflux condition to carry out nano-silica chloride. In this reaction, OH functional groups of silica gel were replaced by Cl atoms of thionyl chloride. Then, nano-silica chloride, which is dried, reacted with DBN in *n-hexane* under reflux condition. The chlorine atoms in nano-silica chloride were replaced with N-nucleophiles in DBN (Scheme1).

**Fig. 2:**
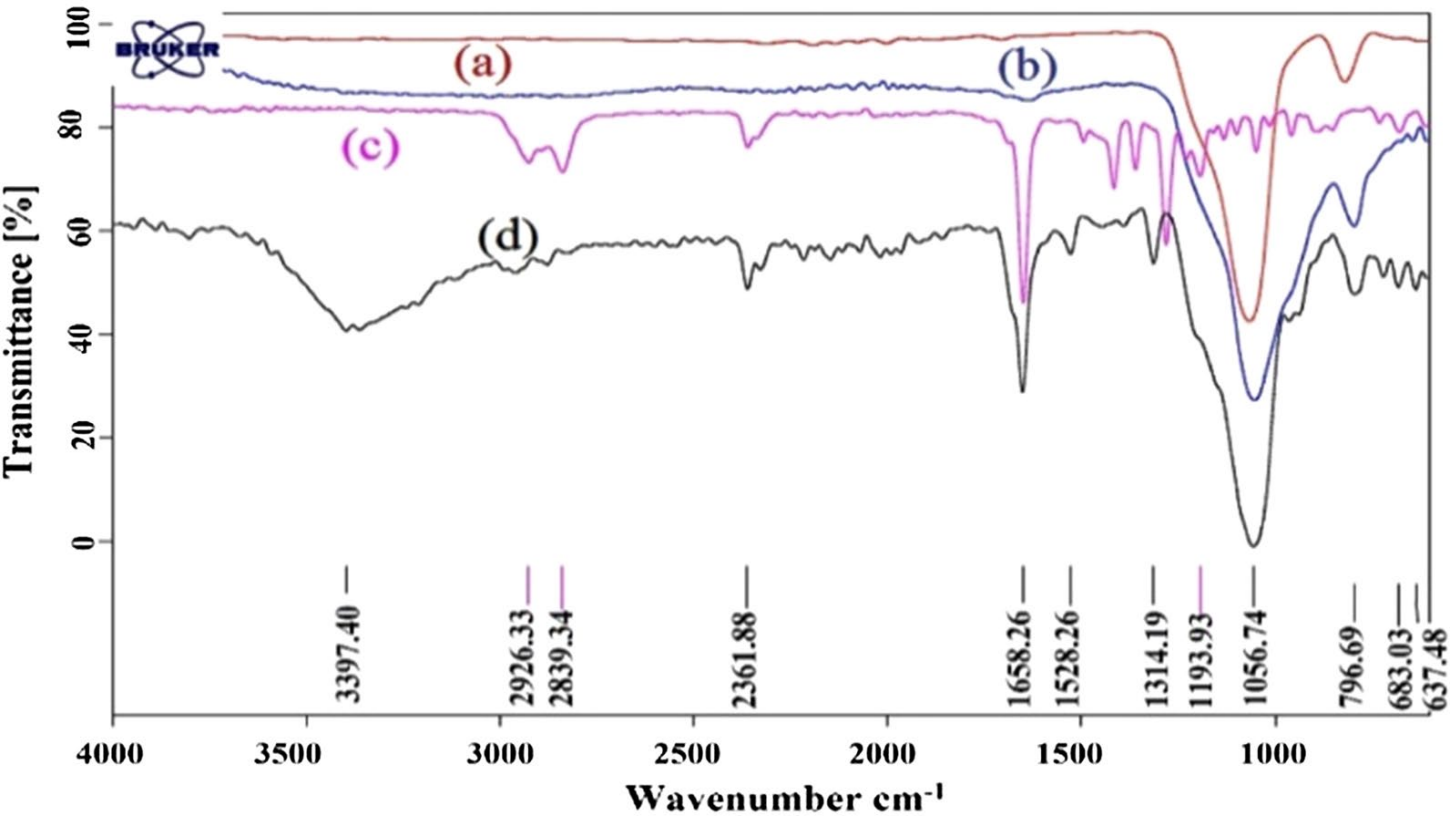
FT-IR spectra of **a** Nano-SiO_2_, **b** nano-silica chloride, **c** DBN, **d** nano-SiO_2_/DBN

Figure 2a–c shows the FT-IR spectra of the synthesized materials. Figure 2d shows absorption band at 3397 cm^−1^ which is due to the SiO–H stretching vibration, 1652 cm^−1^ for the C=N stretching vibration and 1056 cm^−1^ for Si–O stretching vibration and 796 cm^−1^ due to the Si–O–Si bending vibrational mode.

**Fig. 3:**
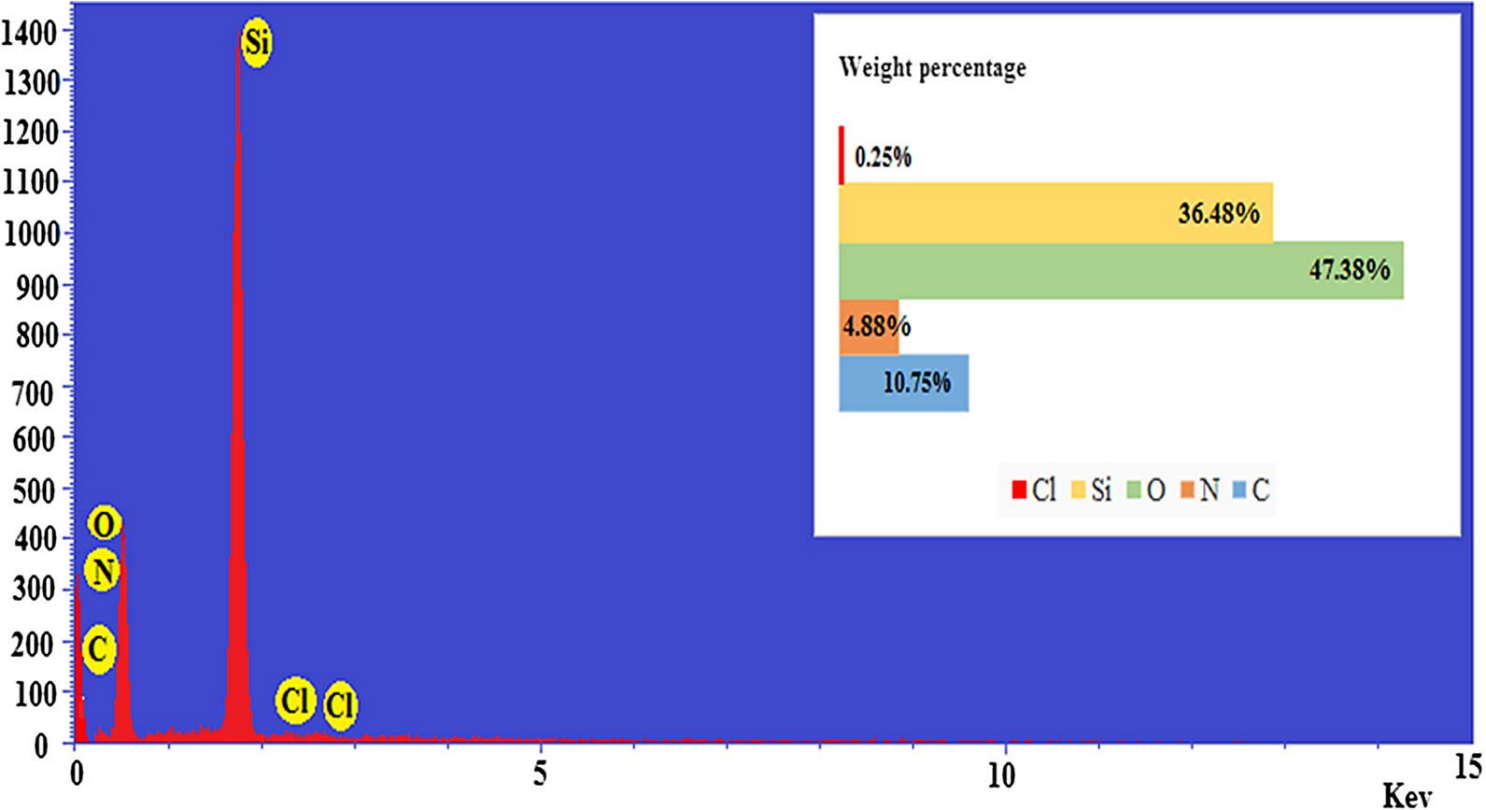
The EDX spectra of nano-SiO_2_/DBN

Energy-dispersive X-ray spectroscopy (EDS) was used to determine the percentage of elements in nano-SiO_2_/DBN (Fig. 3). The percentage of C, N, O, Si and Cl in nano-SiO_2_/DBN was 10.75, 4.88, 47.38, 36.48 and 0.25 respectively.

**Fig. 4:**
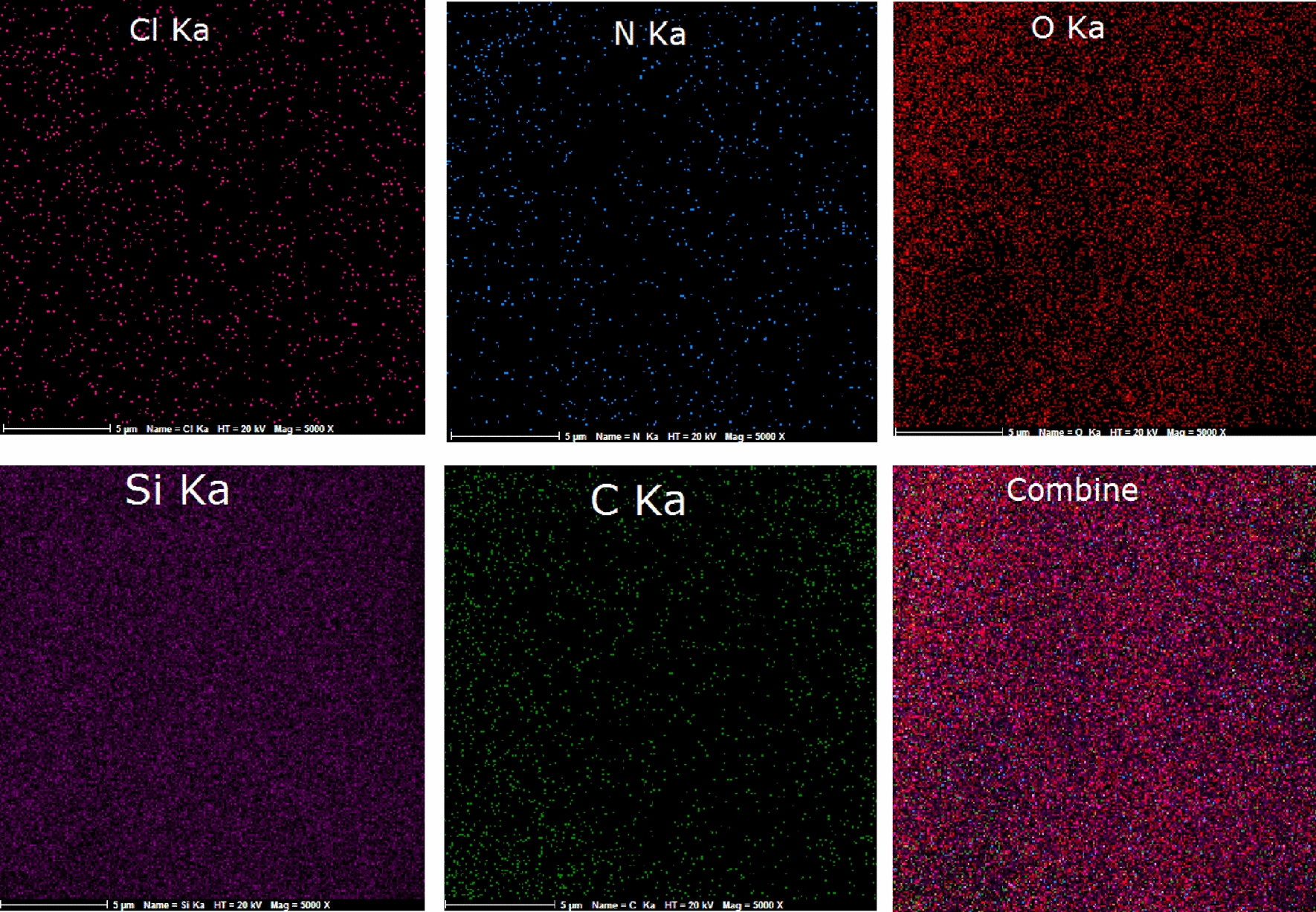
EDX-map of elements in the structure of nano-SiO_2_/DBN

The EDX-map of elements in the structure of nano-SiO_2_/DBN (Fig. 4) displays homogenous distribution of elements in catalyst.

**Fig. 5:**
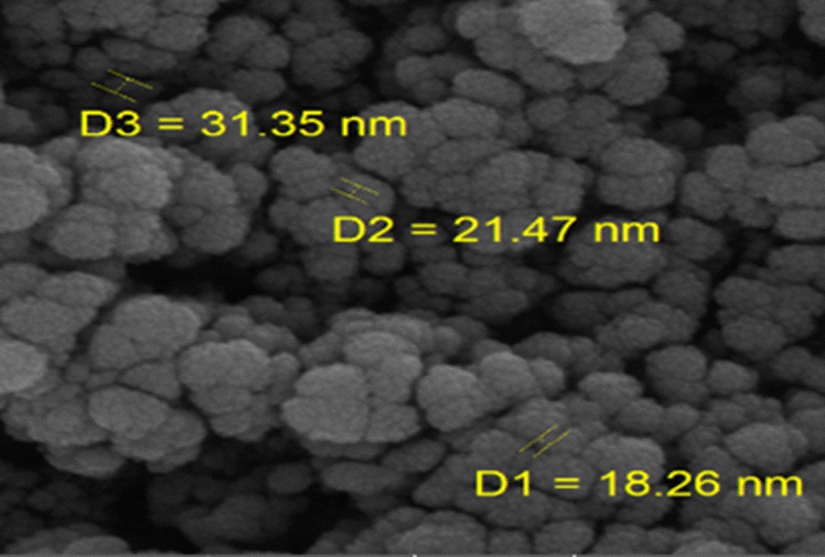
FESEM images of nano-SiO_2_/DBN

The particle size of nano-SiO_2_/DBN was studied using field emission scanning electron microscopy (FESEM) and found to be less than 50 nm (Fig. 5).

**Fig. 6:**
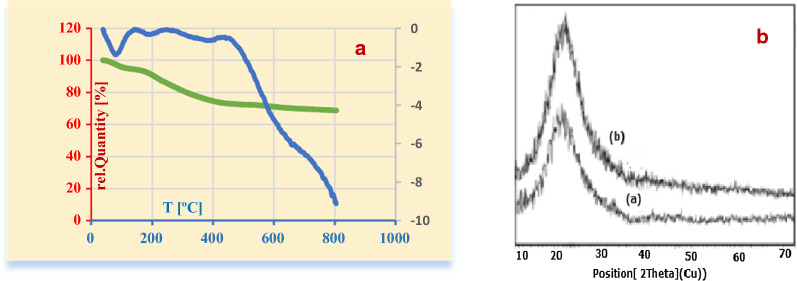
(**A**) TGA patterns of nano-SiO_2_/DBN and (**B**) XDR patterns of a) nano-SiO_2_ and b) nano-SiO_2_/DBN

TGA analysis is shown in Fig. 6A, which exhibits the stability of the nano-SiO_2_/DBN as nano-catalyst which can be used up to 120 °C. The weight loss (4.2%) below 100 °C is likely due to the loss of catalyst moisture. However, the main decomposition occurs at 165–450 °C (20.7%). Nano-SiO_2_/DBN has noticeably high thermal stability with char yield 68.72% at 800 °C.

Figure 6B display the XRD Patterns of nano-SiO_2_, nano-SiO_2_/DBN in the range of 10–80°. A broad peak (Fig. 6B (a)) is observed at 2θ = 23°, showing the SiO_2_ is amorphous. While, the diffraction pattern of the nano-SiO_2_/DBN (Fig. 6B (b)) indicated peak at 2θ = 23.525° with FWHM = 2.3616. According to Scherrer equation, the particle size of catalyst is 3.4 nm.

**Fig. 7:**
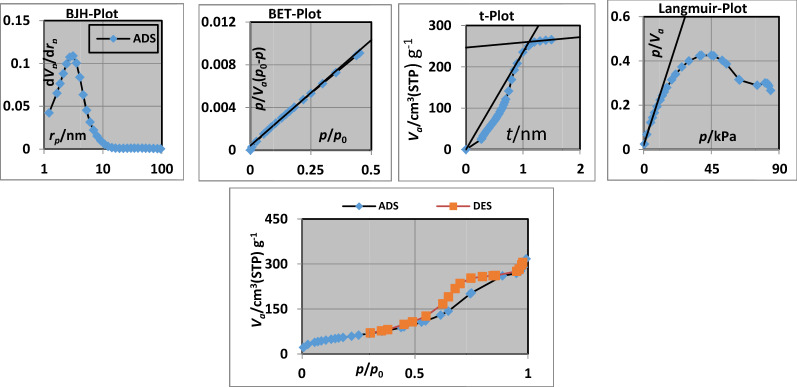
**a** BJH plot, **b** BET (Brunauer–Emmett–Teller) plot, **c** t-plot, **d** Langmuir plot and **e** adsorption/desorption isotherm of nano-SiO_2_/DBN

Figure 7 shows (a) BJH plot, (b) BET (Brunauer–Emmett–Teller) plot, (c) t-plot, (d) Langmuir plot and (e) Adsorption/desorption isotherm of nano-SiO_2_/DBN. The obtained data of BET, Langmuir, t and BJH plots were summarized in Table 1.

**Table 1:** The summerized data of BET, Langmuir, t and BJH plots

BET plot		
*V*_*m*_	49.439	[cm^3^(STP) g^−1^]
a_s,BET_	215.18	[m^2^ g^−1^]
*C*	48.241	
Total pore volume (*p*/*p*_0_ = 0.990)	0.4823	[cm^3^ g^−1^]
Mean pore diameter	8.966	[nm]

Langmuir plot		
Vm	47.372	[cm^3^(STP) g^−1^]
a_s,Lang_	206.18	[m^2^ g^−1^]
B	1.1678	

t plot		
Plot data	Adsorption branch	
a_1_	362.85	[m^2^ g^−1^]
V_1_	0	[cm^3^ g^−1^]
a_2_	19.303	[m^2^ g^−1^]
V_2_	0.3824	[cm^3^ g^−1^]
2t	2.2126	[nm]

BJH plot		
Plot data	Adsorption branch	
V_p_	0.504	[cm^3^ g^−1^]
*r*_*p,peak*_(*Area*)	3.1	[nm]
a_p_	290.06	[m^2^ g^−1^]

To optimize the reaction conditions in the synthesis of tetrahydrobenzo[*b*]pyran, the one-pot three-component condensation reaction of 4-chlorobenzaldehyde, dimedone and malononitrile was investigated, as model reaction, for various factors such as the amount of nano-SiO_2_/DBN, time, temperature and solvent (Table 2). Therefore, the best reaction condition was performed using 0.03 g of catalyst in various solvents such as H_2_O, CHCl_3_, MeOH, EtOH and H_2_O/EtOH (Table 2, entries 1‒5). The use of H_2_O/EtOH (1:1) as solvent at 60 °C is the most efficient condition for the model reaction with high yield and short time (Table 2, entry 10). The reaction performed under solvent free conditions, gave a lower yield in comparison with those performed in the solvent (Table 2, entries 6, 7).

**Table 2:** The reaction of malononitrile, 4-chlorobenzaldehyde and dimedone in the presence of nano-SiO_2_/DBN under various conditions


Entry	Conditions	Time (min)	Yield^a^ (%)
	Solvent/temp (°C)/catalyst (g)		
1	H_2_O/EtOH (1:1)/60/‒	180	28
2	H_2_O/60/0.03	30	70
3	EtOH/60/0.03	30	58
4	CHCl_3_/60/0.03	60	50
5	MeOH/60/0.03	60	70
6	‒/60/0.03	120	42
7	‒/80/0.03	90	58
8	H_2_O/EtOH (1:1)/r.t/0.03	15	10
9	H_2_O/EtOH (1:1)/40/0.03	15	58
10	H_2_O/EtOH (1:1)/60/0.03^b^	15	92
11	H_2_O/EtOH (1:1)/60/0.01	50	76
12	H_2_O/EtOH (1:1)/60/0.02	30	80
13	H_2_O/EtOH (1:1)/60/0.04	20	92
14	H_2_O/EtOH (1:1)/60/0.05	35	70

Reaction conditions: malononitrile (1 mmol), 4-chlorobenzaldehyde (1 mmol), dimedone (1 mmol) and nano-SiO_2_/DBN as catalyst

^a^Isolated yield

^b^The modified condition

After determining the optimized condition, the reaction between different aldehydes with dimedone and malononitrile was investigated (Table 3). In result, tetrahydrobenzo[*b*]pyrans were synthesized in good to high yields and short reaction times. The progress of reaction was monitored by TLC continuously. Meanwhile, the aldehydes with electron withdrawing group in 4-position have reacted in lower time with higher yields (Table 3, entries 2, 5, 8, 9, 10). The aldehydes with a substitution group in 2-position, have steric hindrance which caused longer reaction time (Table 3, entries 3, 4, 5) (Additional file 1).

**Table 3:** Synthesis of tetrahydrobenzo[*b*]pyran in the presence of nano-SiO_2_/DBN at 60 ºC in H_2_O/EtOH (1:1)^a^


Entry	R^a^	Product	Time (min)	Yield (%)^b^	M.P (°C) found	M.P (°C) reported (refs.)
1	C_6_H_5_-	**4a**	20	85	232‒234	234‒235 [22]
2	4-Cl-C_6_H_4_-	**4b**	15	92	214‒216	215‒216 [22]
3	2-Cl–C_6_H_4_-	**4c**	35	81	217‒218	218‒219 [23]
4	2,6-Cl_2_-C_6_H_3_-	**4d**	50	79	245‒247	250‒252 [23]
5	4-NO_2_-C_6_H_4_-	**4e**	15	89	181‒183	181‒182 [27]
6	3-NO_2_-C_6_H_4_-	**4f**	20	91	215‒217	217‒218 [27]
7	2-NO_2_-C_6_H_4_-	**4 g**	35	80	232‒234	233‒234 [22]
8	4-Br-C_6_H_4_-	**4 h**	30	89	199‒201	197‒201 [13]
9	4-F-C_6_H_4_-	**4i**	40	91	192‒194	191‒193 [15]
10	4-CN-C_6_H_4_-	**4j**	25	85	231‒233	226‒228 [24]
11	4-OCH_3_-C_6_H_4_-	**4 k**	25	70	208‒210	208‒212 [23]
12	3,4-(OCH_3_)_2_-C_6_H_3_-	**4 l**	50	82	175‒176	206‒208 [25]
13	4-OH-C_6_H_4_-	**4 m**	40	78	217‒219	214‒216 [23]
14	4-(CH_3_)_2_CH-C_6_H_4_-	**4n**	30	90	197‒199	203‒207 [13]
15	4-CO_2_CH_3_-C_6_H_4_-	**4o**	15	78	257‒259	259‒260 [27]
16	1,4-Phenylene	**4p**	50	91	285 (d)^c^	270 (d) [26]
17	2-Furyl-	**4q**	25	84	216‒218	217‒219 [23]
18	Pentyl-	**4r**	20	83	162‒164	164‒165 [27]
19	Styryl-	**4 s**	30	90	217‒219	218‒218 [23]

4a-s are the synthesized tetrahydrobenzo[b]pyrans with different R

^a^Reaction conditions: malononitrile (1 mmol), aldehyde (1 mmol), dimedone (1 mmol) and nano-SiO_2_/DBN (0.03 g)

^b^Isolated yield

^c^Decomposed

As shown in Table 4, performance of synthesized catalyst compared to nano-SiO_2_, DBN and previously reported catalysts. Nano-SiO_2_/DBN can be presented as an efficacious one, among others, catalyst in terms of reaction time and yields. There are many privileges in this regard simple procedure, nontoxic solvent and mild reaction conditions. DBN is a good catalyst for this reaction, but is not a heterogeneous recoverable catalyst.

**Table 4:** Comparison of nano-SiO_2_/DBN catalyst with some other catalyst for the synthesis of **4b**

Entry	Conditions	Time (min)	Yield^b^ (%) (refs.)
	Solvent/temp (ºC)/catalyst		
1	CH_2_Cl_2_/60/SiO_2_/NH_2_OAc	360	90 [28]
2	H_2_O/80/Fe_3_O_4_@SiO_2_/DABCO	25	90 [29]
3	EtOH /reflux/l-Proline	360	87 [30]
4	EtOH /reflux/SO_4_^2−^/MCM-41	60	80 [31]
5	H_2_O/reflux/DABCO	150	68 [32]
6	H_2_O/120/SDS	120	85 [33]
7	H_2_O/60/Thiourea dioxide	13	93 [34]
8	H_2_O/EtOH/60/catalyst^a^	15	92 (this work)^c^
9	H_2_O/EtOH/60/nano-SiO_2_	15	40 (this work)
10	H_2_O/EtOH/DBN	15	93 (this work)

^a^Nano-SiO_2_/DBN

^b^Isolated yield

^c^The modified condition

**Scheme 2:**
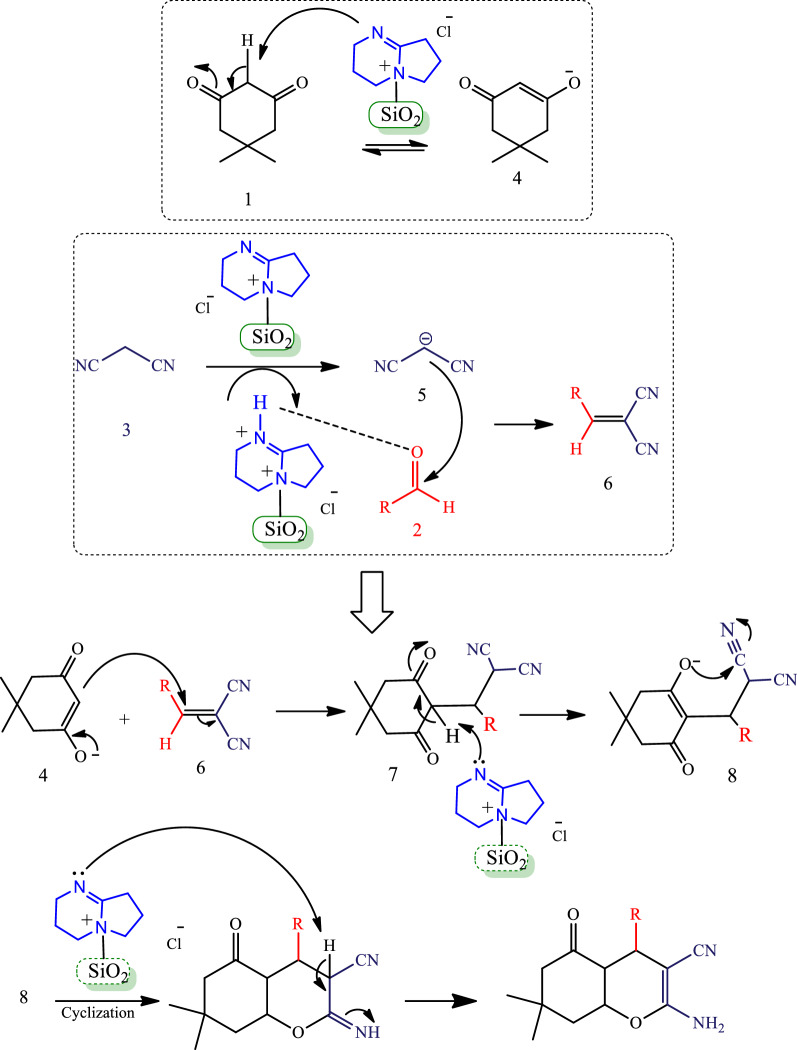
The plausible mechanism for the synthesis of tetrahydrobenzo[*b*]pyran derivatives

A suggested mechanism for synthesis of tetrahydrobenzo[*b*]pyran derivatives by using nano-SiO_2_/DBN is illustrated in Scheme 2. Initially, the nano-SiO_2_/DBN catalyst activates both the methylene group **5** and the carbonyl group **1**. After, the Knoevenagal condensation reaction between the malononitrile and aldehyde in existence of basic catalyst forms the intermediate **6**. Then, the Michael addition of enol **4** and intermediate **6** is performed to produce the intermediate **7**. Finally, the product is formed by cyclization and tautomerization of the intermediate **8**.

**Fig. 8:**
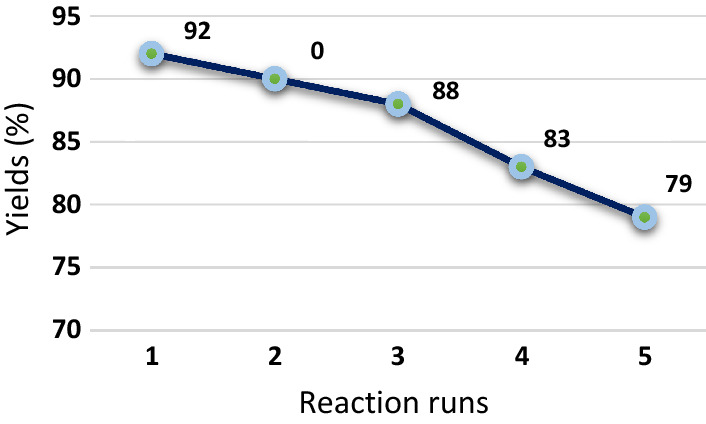
Reusability of the nano-SiO_2_/DBN catalyst.

The reusability of the nano-SiO_2_/DBN was investigated. After completion of the reaction, the nanocatalyst was separated and washed with some EtOH, then dried at 70 °C. The catalyst was regained in good yields and catalyst was used in the synthesis of tetrahydrobenzo[*b*]pyran for five times (Fig. 8).

## Conclusion

We have reported one-pot three component condensation reaction of various aldehydes, malononitrile and dimedone at 60 °C under mild conditions. The novel synthesis has been explored of tetrahydrobenzo[*b*]pyran derivatives in the presence nano-SiO_2_/DBN as a heterogeneous nanocatalyst. The synthesized nanocatalyst was characterized by FT-IR, XRD, FESEM, TGA, EDS and BET analysers. The advantages of this method are summarized in the following orders, inexpensive, recyclability and reusability of the catalyst, easy work-up and good yield of the products, the use of relatively environmentally benign solvents, short reaction times and simplicity experimental of the procedure.

## Supplementary Information

**Additional file 1.** Spectroscopic data for the synthesized tetrahydrobenzo[b]pyran derivatives.

## Data Availability

All data generated or analyzed during this study are included in this published article.

## Abbreviations

DBN: 1,5-Diazabicyclo[4.3.0]non-5-en
MCRs: Multi-component reactions
EtOH: Ethanol
MeOH: Methanol
CH_3_Cl: Chloroform
DMSO: Dimethyl sulfoxide
FESEM: Field emission scanning electron microscope
FT-IR: Fourier transform infrared
XRD: X-ray diffraction
EDX: Energy-dispersive X-ray
TGA: Thermo gravimetric analysis
NMR: Nuclear magnetic resonance
TLC: Thin layer chromatography
EDS: Dispersive X-ray spectroscopy

## References

[1] Domling A, Wang W, Wang K (2012). Chemistry and biology of multicomponent reactions. Chem Rev.

[2] Shaabani A, Maleki A, Rezayan AH, Sarvary A (2011). Recent progress of isocyanide-based multicomponent reactions in Iran. Mol Divers.

[3] Shaabani A, Amini MM, Ghasemi S, Ghadari R, Rezayan AH, Fazaeli Y, Feizi S (2010). Pyridine-functionalized MCM-41 as an efficient and recoverable catalyst for the synthesis of pyran annulated heterocyclic systems. Chem Pharm Bull.

[4] Banerjee S, Horn A, Khatri H, Sereda G (2011). A green one-pot multicomponent synthesis of 4*H*-pyrans and polysubstituted aniline derivatives of biological, pharmacological, and optical applications using silica nanoparticles as reusable catalyst. Tetrahedron lett.

[5] Banerjee S, Sereda G (2009). One-step, three-component synthesis of highly substituted pyridines using silica nanoparticle as reusable catalyst. Tetrahedron Lett.

[6] Kumar D, Sharma P, Singh H, Nepali K, Gupta GK, Jain SK, Ntie-Kang F (2017). The value of pyrans as anticancer scaffolds in medicinal chemistry. RSC Adv.

[7] Latif N, Mishriky N, Assad FM (1982). Carbonyl and thiocarbonyl compounds. XIX. Intramolecular cyclization of (2-nitroetheny1)aryl*N*-arylcarbamates: synthesis of newer series of 3,4-dihydro-2H-1, 3-oxazin-2-ones and their antimicrobial activities. Aust J Chem.

[8] Chylińska JB, Urbański T, Mordarski M (1963). Dihydro-1,3-oxazine derivatives and their antitumor activity. J Med Chem.

[9] Patil AD, Freyer AJ, Eggleston DS, Haltiwanger RC, Bean MF, Taylor PB, Bartus HR (1993). The inophyllums, novel inhibitors of HIV-1 reverse transcriptase isolated from the Malaysian tree *Calophyllum inophyllum* Linn. J Med Chem.

[10] Bisht SS, Jaiswal N, Sharma A, Fatima S, Sharma R, Rahuja N, Tripathi RP (2011). A convenient synthesis of novel pyranosyl homo-C-nucleosides and their antidiabetic activities. Carbohydr Res.

[11] Schiller R, Tichotová L, Pavlík J, Buchta V, Melichar B, Votruba I, Pour M (2010). 3,5-Disubstituted pyranone analogues of highly antifungally active furanones: conversion of biological effect from antifungal to cytostatic. Bioorg Med Chem Lett.

[12] Maleki H, Rakhtshah J, Shaabani B (2020). Effective one-pot synthesis of tetrahydrobenzo[*b*]pyran derivatives using nickel Schiff-base complex immobilized on iron oxide nanoparticles. Appl Organomet Chem.

[13] Zarei A, Yarie M, Zolfigol MA, Niknam K (2020). Synthesis of a novel bifunctional oxyammonium-based ionic liquid: application for the synthesis of pyrano[*4,3-b*]pyrans and tetrahydrobenzo[*b*]pyrans. J Chin Chem Soc.

[14] Hiremath PB, Kantharaju K (2020). An efficient and facile synthesis of 2-amino-4H-pyrans & tetrahydrobenzo[*b*]pyrans catalysed by WEMFSA at room temperature. ChemistrySelect.

[15] Sharma H, Srivastava S (2018). Anion–cation co-operative catalysis by artificial sweetener saccharine-based ionic liquid for sustainable synthesis of 3,4-dihydropyrano[*c*]chromenes, 4,5-dihydropyrano[*4,3-b*]pyran and tetrahydrobenzo[*b*]pyrans in aqueous medium. RSC Adv.

[16] Niya HF, Hazeri N, Kahkhaie MR, Maghsoodlou MT (2020). Preparation and characterization of MNPs–PhSO_3_H as a heterogeneous catalyst for the synthesis of benzo[*b*]pyran and pyrano[*3,2-c*]chromenes. Res Chem Intermed.

[17] Singh P, Yadav P, Mishra A, Awasthi SK (2020). Green and mechanochemical One-Pot multicomponent synthesis of bioactive 2-amino-4*H*-benzo[*b*]pyrans via highly efficient amine-functionalized SiO_2_@Fe_3_O_4_ nanoparticles. ACS Omega.

[18] Sayahi MH, Gorjizadeh M, Meheiseni M, Sayyahi S (2020). One-pot multi-component process for the synthesis of 4-azaphenanthrene-3,10-dione,1,8-dioxo-octahydroxanthene and tetrahydrobenzo[*b*]pyran derivatives catalyzed by the deep eutectic solvent choline chloride-oxalic acid. Z Naturforsch B.

[19] Hou F, Zheng W, Yousefi N (2020). Design, characterization and application of the SCMNPs@PC/VB1-Zn as a green and recyclable biocatalyst for synthesis of pyrano[*2,3-c*]pyrazole and 4*H*-benzo-[*b*]-pyran derivatives. Bull Chem React Eng Catal.

[20] Adibian F, Pourali AR, Maleki B, Baghayeri M, Amiri A (2020). One-pot synthesis of dihydro-1H-indeno [1,2-b] pyridines and tetrahydrobenzo[*b*]pyran derivatives using a new and efficient nanocomposite catalyst based on *N*-butylsulfonate-functionalized MMWCNTs-D-NH2. Polyhedron.

[21] Shirzaei M, Mollashahi E, Maghsoodlou MT, Lashkari M (2020). Application of chlorophyll extracted from spinach as a green and affordable catalyst for the synthesis of tetrahydrobenzo[*b*]pyran and pyrano[*c*]chromene. Org Chem Res.

[22] Gao S, Tsai CH, Tseng C, Yao CF (2008). Fluoride ion catalyzed multicomponent reactions for efficient synthesis of 4*H*-chromene and *N*-arylquinoline derivatives in aqueous media. Tetrahedron.

[23] Khazaei A, Gholami F, Khakyzadeh V, Moosavi-Zare AR, Afsar J (2015). Magnetic core–shell titanium dioxide nanoparticles as an efficient catalyst for domino Knoevenagel–Michael-cyclocondensation reaction of malononitrile, various aldehydes and dimedone. RSC Adv.

[24] Maleki B, Nasiri N, Tayebee R, Khojastehnezhad A, Akhlaghi HA (2016). Green synthesis of tetrahydrobenzo[*b*]pyrans, pyrano[*2,3-c*]pyrazoles and spiro[indoline-3,4′-pyrano[*2,3-c*]pyrazoles catalyzed by nano-structured diphosphate in water. RSC Adv.

[25] Ren Y, Zhang W, Lu J, Gao K, Liao X, Chen X (2015). One-pot synthesis of tetrahydro-4 H-chromenes by supramolecular catalysis in water. RSC adv.

[26] Hasaninejad A, Golzar N, Beyrati M, Zare A, Doroodmand MM (2013). Silica-bonded 5-n-propyl-octahydro-pyrimido[*1*,*2-a*]azepinium chloride (SB-DBU) Cl as a highly efficient, heterogeneous and recyclable silica-supported ionic liquid catalyst for the synthesis of benzo[*b*]pyran, bis(benzo[*b*]pyran) and spiro-pyran derivatives. J Mol Catal A Chem.

[27] Khazdooz L, Zarei A, Ahmadi T, Aghaei H, Golestanifar L, Sheikhan N (2018). Highly efficient and environmentally benign method for the synthesis of tetrahydrobenzo[*b*]pyrans using Ca_9.5_Mg_0.5_(PO_4_)_5.5_(SiO_4_)_0.5_F_1.5_ as a new bio-and nanocatalyst with Brønsted base and Lewis acid properties. Res Chem Intermed.

[28] Gupta R, Gupta M, Paul S, Gupta R (2009). Silica supported ammonium acetate: an efficient and recyclable heterogeneous catalyst for Knoevenagel condensation between adehydes or ketones and active methylene group in liquid phase. Bull Korean Chem Soc.

[29] Kiasat AR, Davarpanah J (2013). Fe_3_O_4_@silica sulfuric acid nanoparticles: an efficient reusable nanomagnetic catalyst as potent solid acid for one-pot solvent-free synthesis of indazolo[*2*,*1-b*]phthalazine-triones and pyrazolo[*1*,*2-b*]phthalazine-diones. J Mol Catal A Chem.

[30] Karade NN, Budhewar VH, Shinde SV, Jadhav WN (2007). l-Proline as an efficient organo-catalyst for the synthesis of polyhydroquinoline via multicomponent Hantzsch reaction. Lett Org Chem.

[31] Abdollahi-Alibeik M, Nezampour F (2013). Synthesis of 4*H*-benzo[*b*]pyrans in the presence of sulfated MCM-41 nanoparticles as efficient and reusable solid acid catalyst. React Kinet Mech Catal.

[32] Lohar T, Kumbhar A, Barge M, Salunkhe R (2016). DABCO functionalized dicationic ionic liquid (DDIL): a novel green benchmark in multicomponent synthesis of heterocyclic scaffolds under sustainable reaction conditions. J Mol Liq.

[33] Mansoor SS, Logaiya K, Aswin K, Sudhan PN (2015). An appropriate one-pot synthesis of 3,4-dihydropyrano[*c*]chromenes and 6-amino-5-cyano-4-aryl-2-methyl-4*H*-pyrans with thiourea dioxide as an efficient, reusable organic catalyst in aqueous medium. J Taibah Univ Sci.

[34] Mehrabi H, Abusaidi H (2010). Synthesis of biscoumarin and 3,4-dihydropyrano[*c*]chromene derivatives catalysed by sodium dodecyl sulfate (SDS) in neat water. J Iran Chem Soc.

