# Supplementary material for: Nano-SiO2/DBN: an efficacious and reusable catalyst for one-pot synthesis of tetrahydrobenzo[b]pyran derivatives
Source: BMC Chem. 2021 May 21;15(1):34. doi: 10.1186/s13065-021-00760-3 (PMC8140504; doi:10.1186/s13065-021-00760-3)
Supplement: Supplementary file 1 — Additional file 1. Spectroscopic data for the synthesized tetrahydrobenzo[b]pyran derivatives. [file 13065_2021_760_MOESM1_ESM.pdf]

Nano-SiO_2/DBN: An efficacious and reusable catalyst for one-pot synthesis of tetrahydrobenzo[*b*]pyran derivatives

Maryam Mehravar[a], Bi Bi Fatameh Mirjalili[a]*, Elaheh Babaei[a], Abdolhamid Bamoniri[b]

[a] Department of Chemistry, College of Science, Yazd University, Yazd, Iran.
[b] Department of Organic Chemistry, Faculty of Chemistry, University of Kashan, Kashan, Iran
E-mail: fmirjalili@yazd.ac.ir

Spectral data for selected compounds

2-Amino-3-cyano-7,7-dimethyl-5-oxo-4-phenyl-4H-5,6,7,8-tetrahydrobenzo[*b*]pyran (Table 3, 4a). Wite solid, mp 232–234 °C. FT-IR (ATR)/υ(cm^{-1}): 3386 (NH_2), 3208 (NH_2), 2198 (C≡N), 1677 (C=O), 1656(C=C), 1212(C–O). ^1H NMR (400 MHz, DMSO-d_6) δ (ppm): 0.94 (s, 3H), 1.02 (s, 3H), 2.10 (d, *J* = 16.0 Hz, 1H), 2.26 (d, *J* = 16.0 Hz, 1H), 2.49–2.55 (m, 2H), 4.15 (s, 1H), 7.00 (s, 2 H, NH_2), 7.11–7.29 (m, 4H).

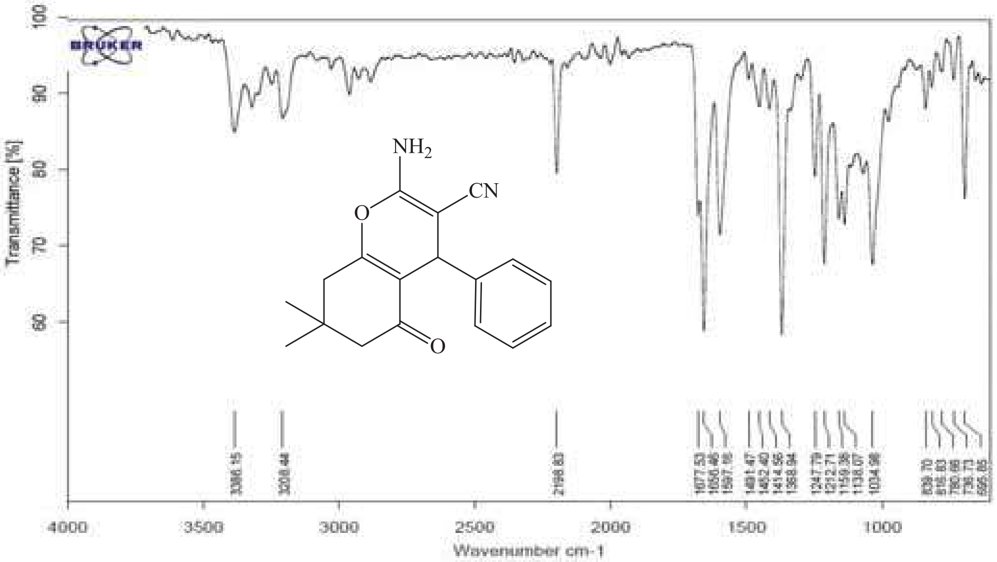

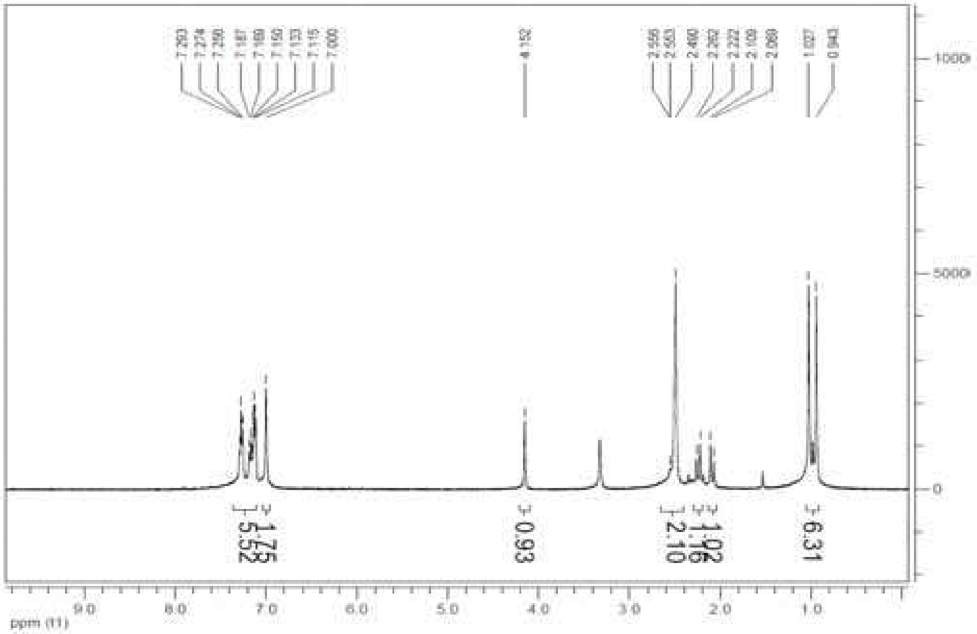

2-Amino-4-(4-chlorophenyl)-3-cyano-7,7-dimethyl-5-oxo-4H-5,6,7,8-tetrahydrobenzo[*b*]pyran (Table 3, 4b) .White solid, mp 214–216 °C**.** FT-IR (ATR)/υ(cm^{-1}): 3422 (NH_2), 3336 (NH_2), 2184 (CN), 1659 (C=O), 1637 (C=C), 1209 (C–O).^{1}H NMR (400 MHz, DMSO-d_6) δ (ppm): 0.95 (s, 3H), 1.01 (s, 3H), 2.10 (d, *J* = 16.0 Hz, 1H), 2.23 (d, *J* = 16.0 Hz, 1H), 2.44–2.54 (m, 2H), 4.18 (s, 1H), 7.08 (s, 2H, NH_2), 7.16 (d, *J* = 7.6 Hz, 2H), 7.33 (d, *J* = 7.6 Hz, 2H). ^{13}C NMR (100 MHz, DMSO-d_6) δ (ppm): 27.31, 28.80, 32.24, 35.60, 50.40, 58.23, 112.80, 120.07, 128.76, 129.59, 131.61, 144.21, 158.96, 163.08, 196.13.

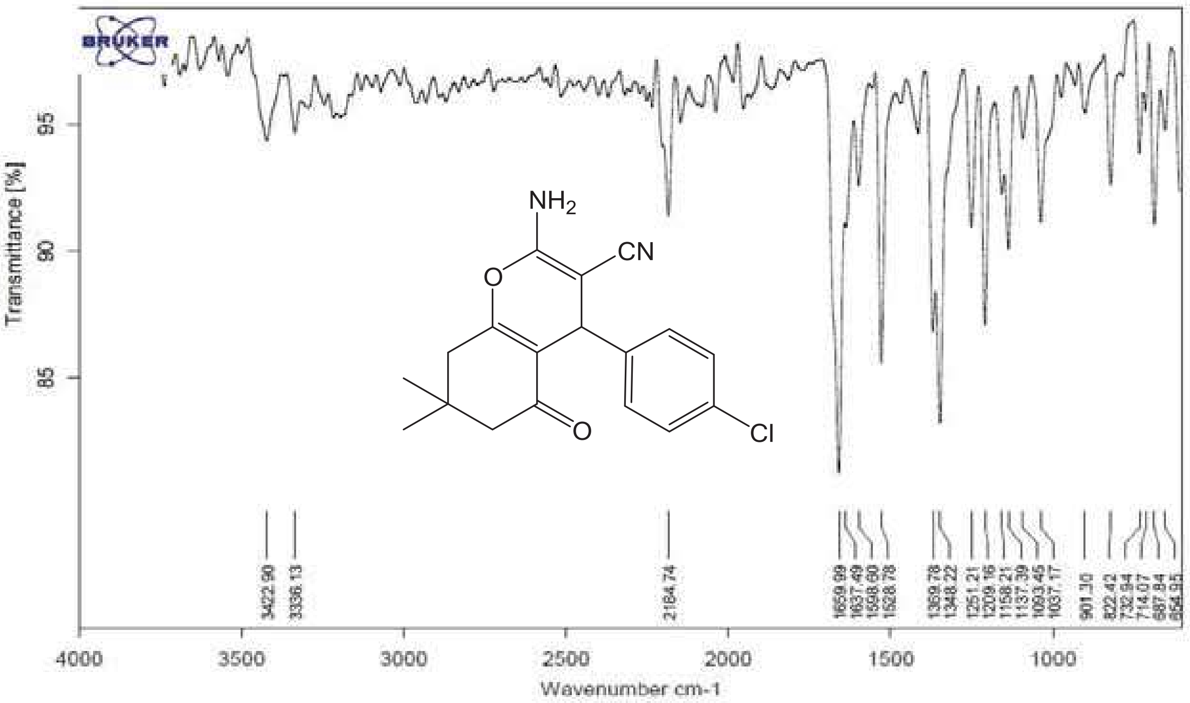

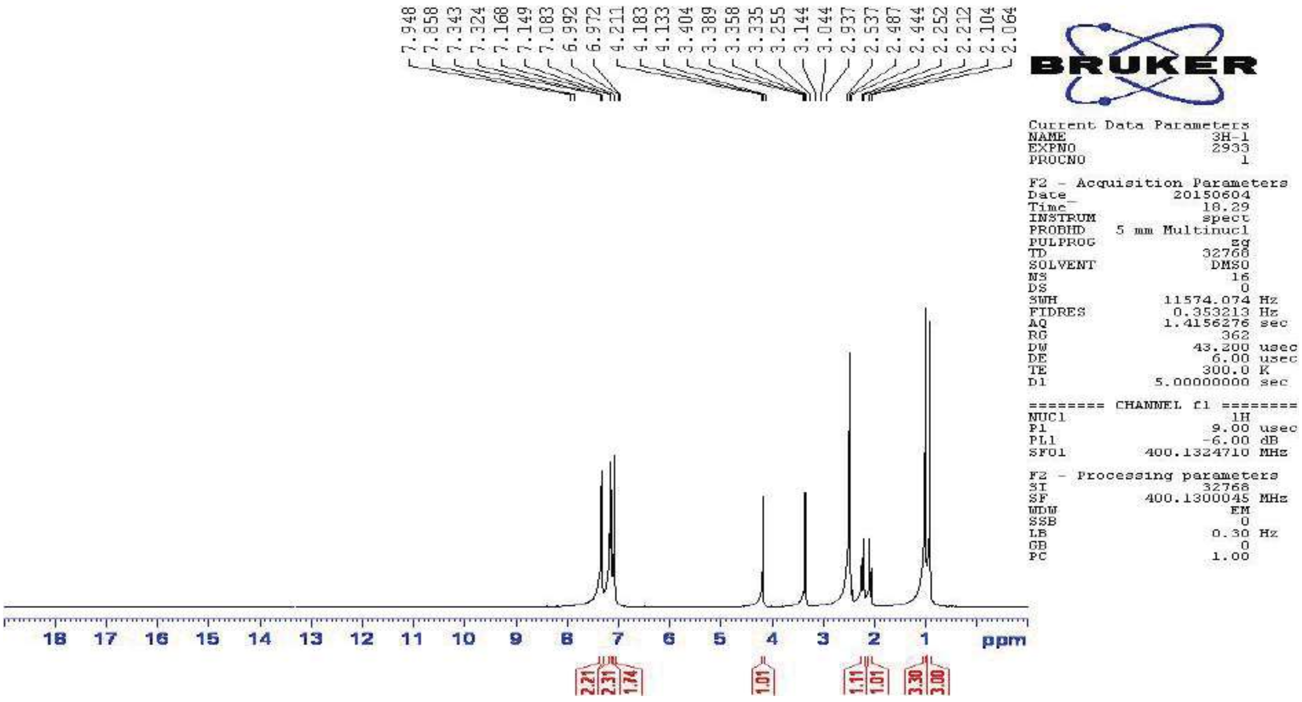

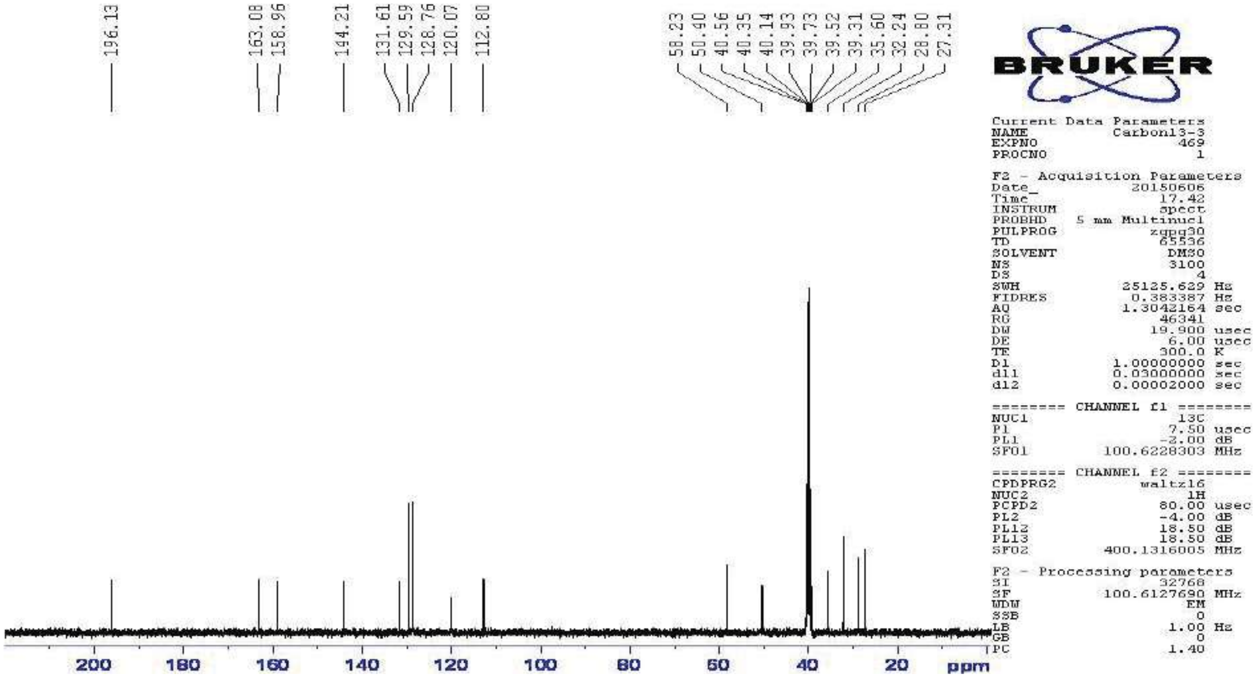

2-Amino-4-(2-chlorophenyl)-3-cyano-7,7-dimethyl-5-oxo-4H-5,6,7,8-tetrahydrobenzo[*b*]pyran (Table 3, 4c). Pale yellow solid, mp 217–218 °C. FT-IR (ATR)/υ(cm^{-1}): 3388 (NH_2), 3327 (NH_2), 2197 (CN), 1653 (C=O), 1654 (C=C), 1214 (C-O); ^1H NMR (400 MHz, DMSO-d_6) δ (ppm): 0.97 (s, 3H), 1.03 (s, 3H), 2.08 (d, *J* = 16.0 Hz, 1H), 2.25 (d, *J* = 16.0 Hz, 1H), 2.45–2.56 (m, 2H), 4.68 (s, 1H), 7.12 (s, 2 H, NH_2), 7.15-7.36 (m, 4H).

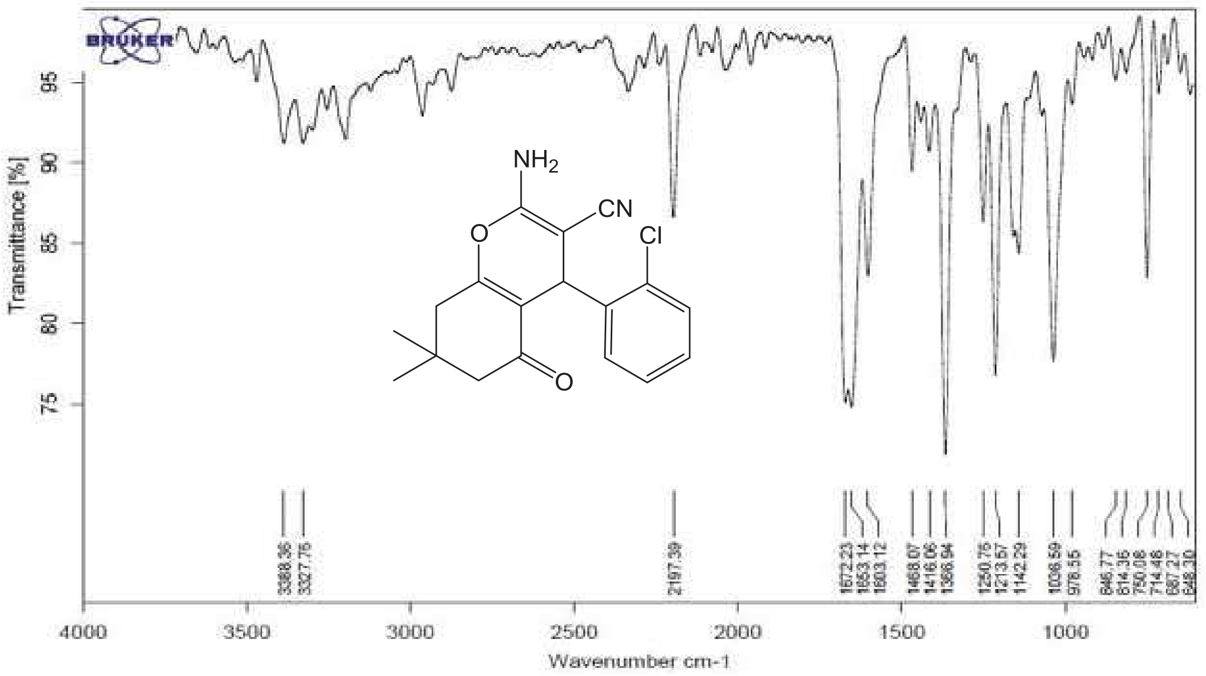

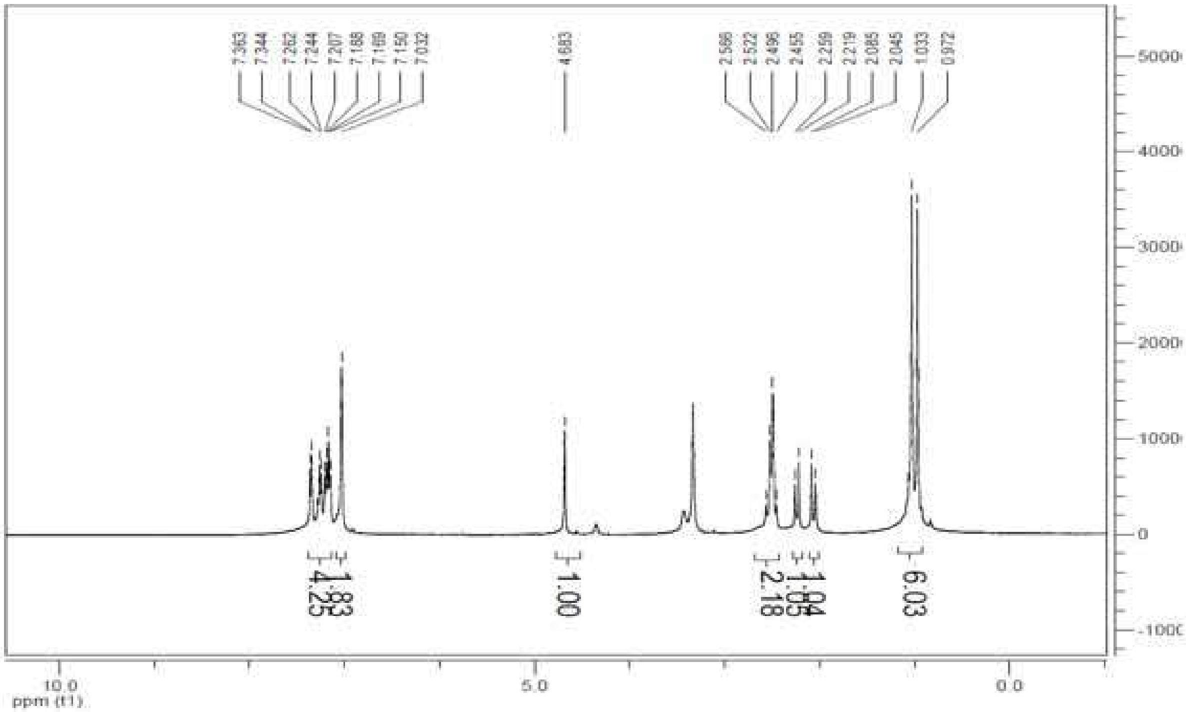

2-Amino-4-(2,6-chlorophenyl)-3-cyano-7,7-dimethyl-5-oxo-4H-5,6,7,8-tetrahydrobenzo[*b*]pyran (Table 3, 4d). White solid, mp 245–247 °C. FT-IR (ATR)/υ(cm^{-1}): 3378 (NH_2), 3322 (NH_2), 2187 (CN), 1674 (C=O), 1631(C=C), 1214 (C-O); ^1H NMR (400MHz, DMSO-d_6) δ (ppm): 0.98 (s, 3H), 1.04 (s, 3H), 2.05 (d, 1H, J = 16.0 Hz), 2.23 (d, 1H, J = 16.0 Hz), 2.35 (d, 1H, J = 16.0 Hz), 2.50 (d, 1H, J =16.0 Hz), 5.19 (s, 1H), 7.09 (s, 2H, NH_2), 7.23 (t, 1H, J = 8.0 Hz), 7.33 (d, 1H, J = 7.2 Hz), 7.43 (d, 1H, J = 6.8 Hz); ^{13}C NMR (100MHz, DMSO-d_6) δ (ppm): 28.9, 32.0, 32.7, 50.3, 56.5, 110.4, 128.9, 129.4, 130.6, 134.5, 136.7, 159.9, 164.2, 196.2.

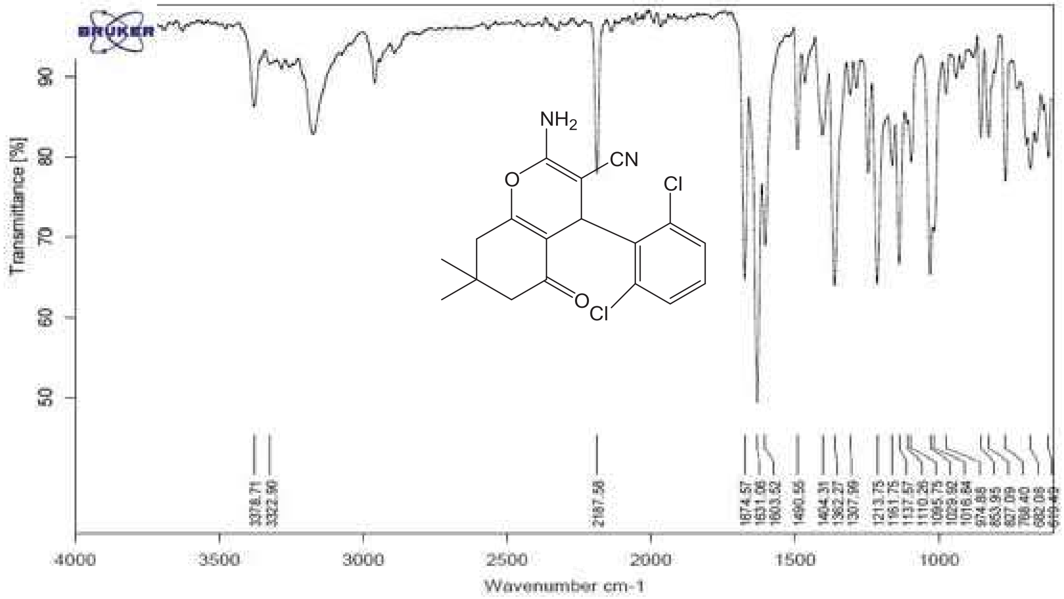

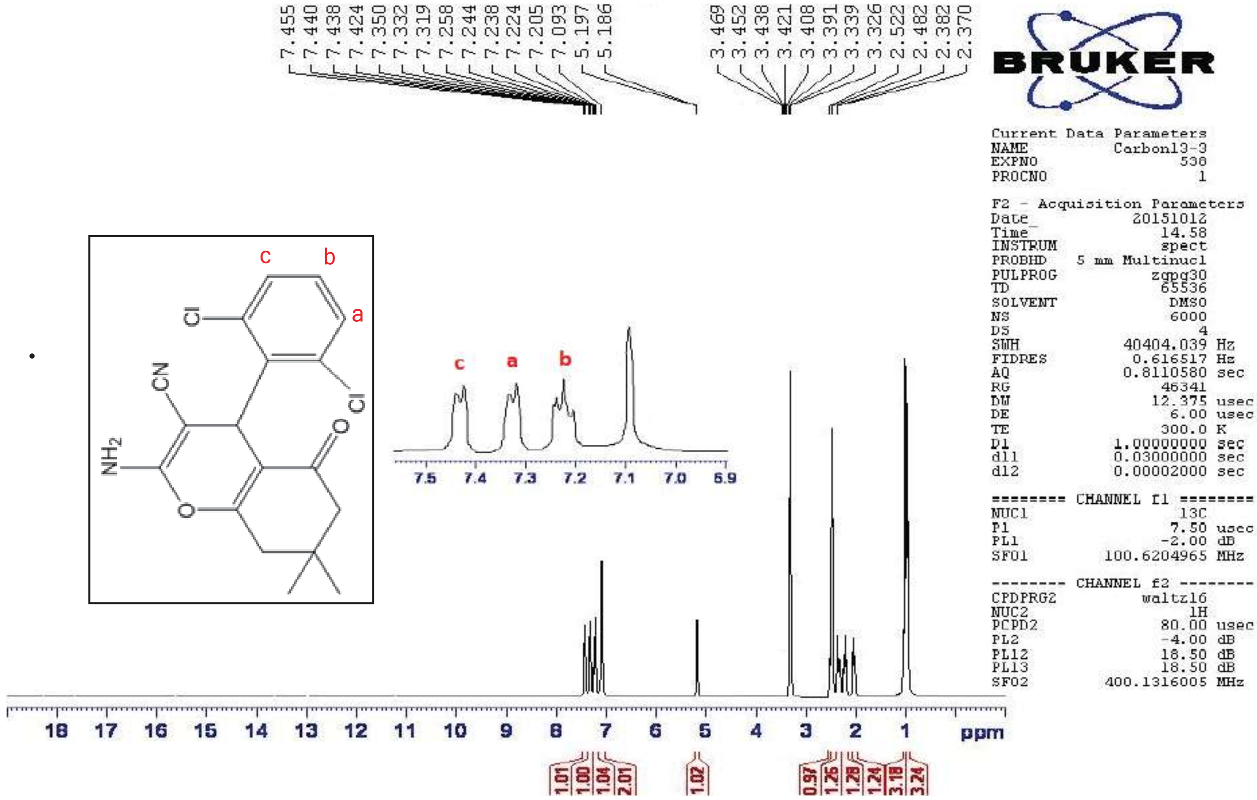

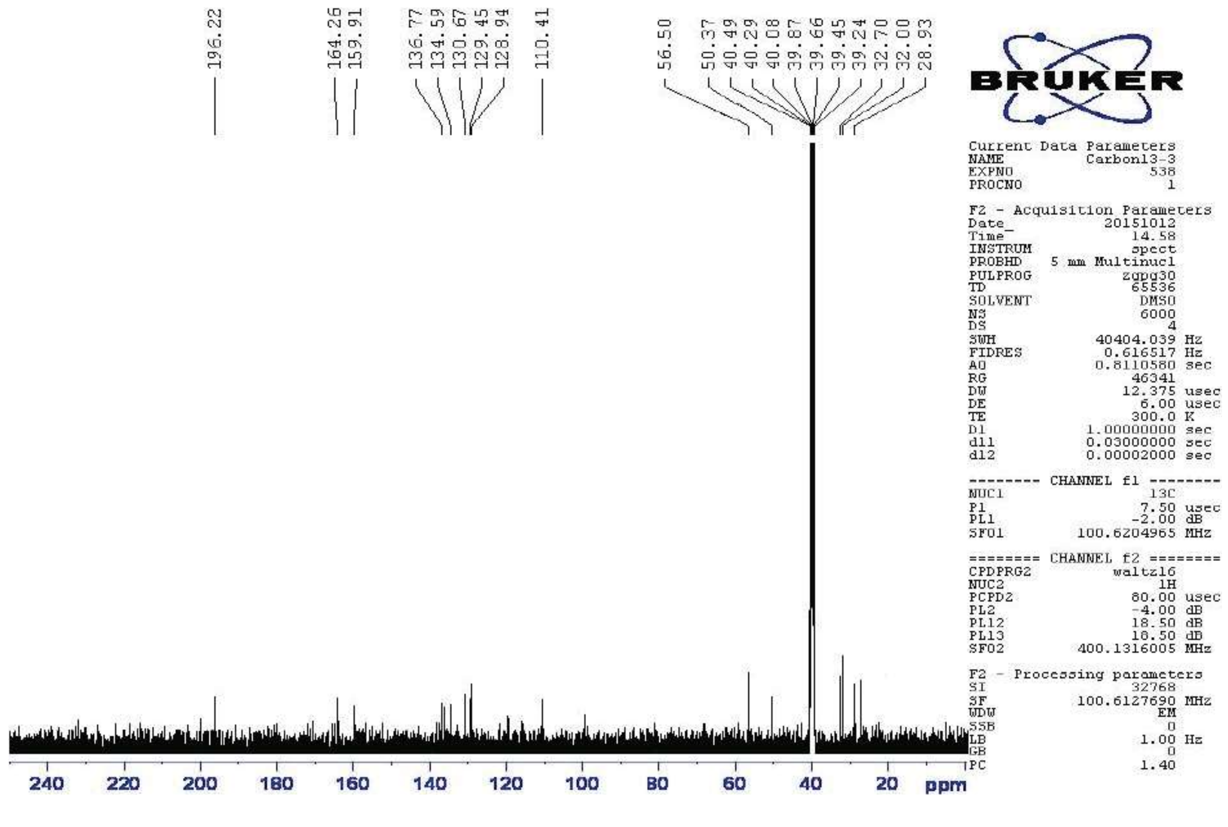

2-Amino -7, 7-dimethyl-4-(4-nitrophenyl)-5-oxo-5, 6, 7, 8-tetrahydrobenzo[*b*]pyran (Table 3, 4e). Yellow solid, mp 181–183 °C. **FT-IR** (ATR)/υ(cm^{-1}): 3407 (NH_2), 3317 (NH_2), 2182 (C≡N),

1668 (C=O), 1627 (C=C), 1519, 1347 (NO_2), 1213 (C–O). **^{1}H NMR** (400MHz, DMSO-d_6) δ (ppm): 0.94 (s, 3H), 1.03 (s, 3H), 2.12 (d, *J* = 16.0 Hz, 1H), 2.27 (d, *J* = 16.0 Hz, 1H), 2.49–2.52 (m, 2H), 4.18 (s, 1H), 7.17 (s, 2H, NH_2), 7.44 (d, *J* = 8 Hz, 2H), 8.173 (d, *J* = 8 Hz, 2H).

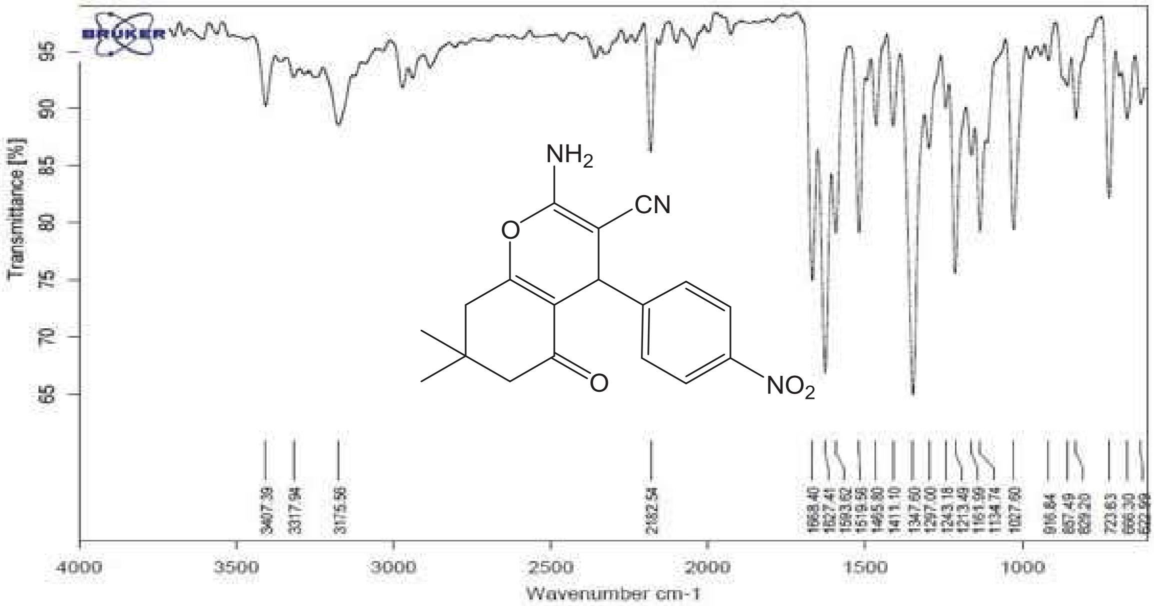

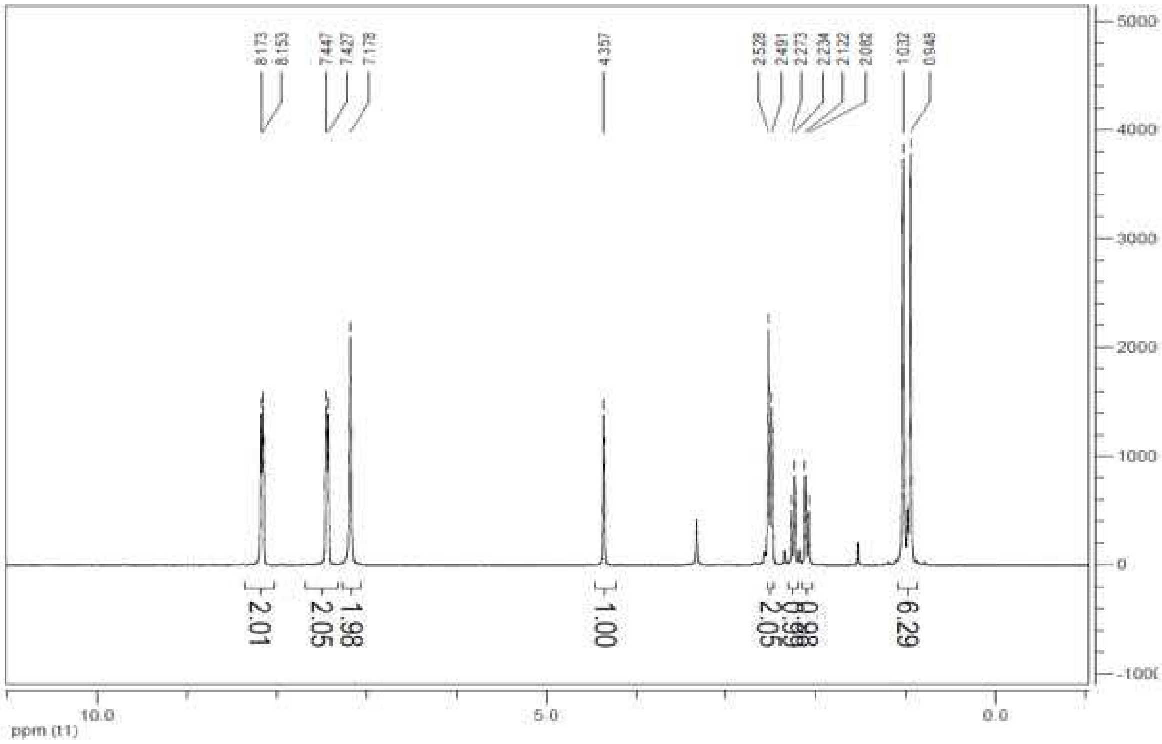

2-Amino -7, 7-dimethyl-4-(3-nitrophenyl)-5-oxo-5, 6, 7, 8-tetrahydrobenzo[*b*]pyran (Table 3, 4f). Yellow solid, mp 215–217 °C. FT-IR (ATR)/υ(cm^{-1}): 3429 (NH_2), 3332 (NH_2), 2185 (CN),

1659 (C=O), 1637 (C=C), 1528, 1348 (NO_2), 1208 (C-O); ^{1}H NMR (400MHz, DMSO-d_6) δ (ppm): 0.96 (s, 3H), 1.04 (s, 3H), 2.12 (d, 1H, J = 16.0 Hz), 2.30 (d, 1H, J = 16.0 Hz), 2.50-2.60 (m, 2H), 4.42 (s, 1H), 7.21 (s, 2H, NH_2), 7.63 (t, 1H, J = 8.0 Hz), 7.68 (d, 1H, J = 8.0 Hz), 7.98(s, 1H), 8.08 (d, 1H, J = 8.0 Hz); ^{13}C NMR(100MHz, DMSO-d_6) δ (ppm): 26.6, 28.3, 31.6, 35.3, 49.8, 57.1, 111.7, 119.3, 121.6, 121.7, 130.0, 134.1, 146.9, 147.7, 158.5, 163.1, 195.7.

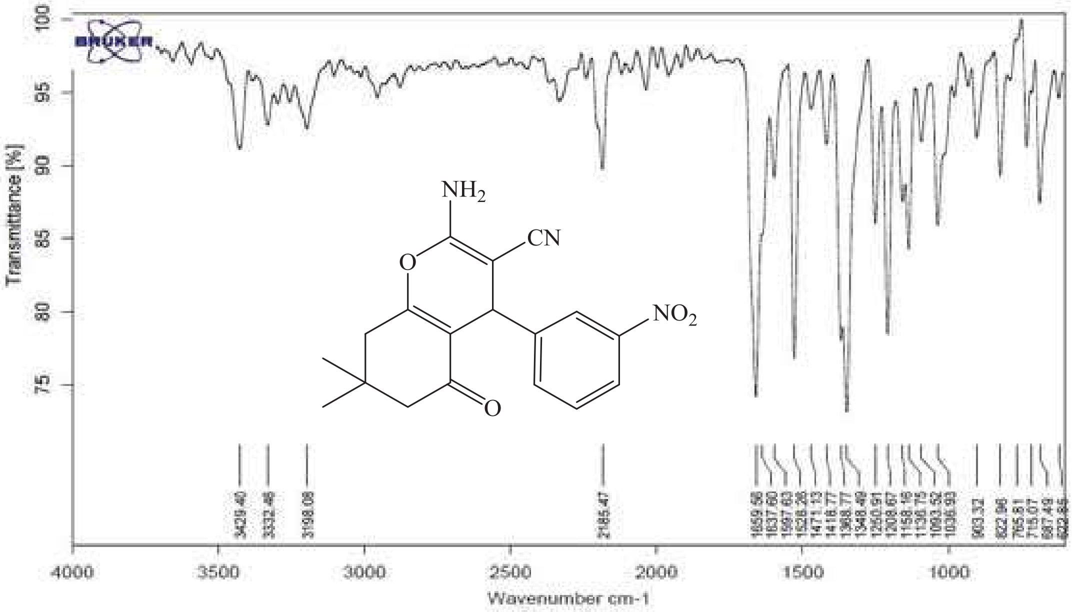

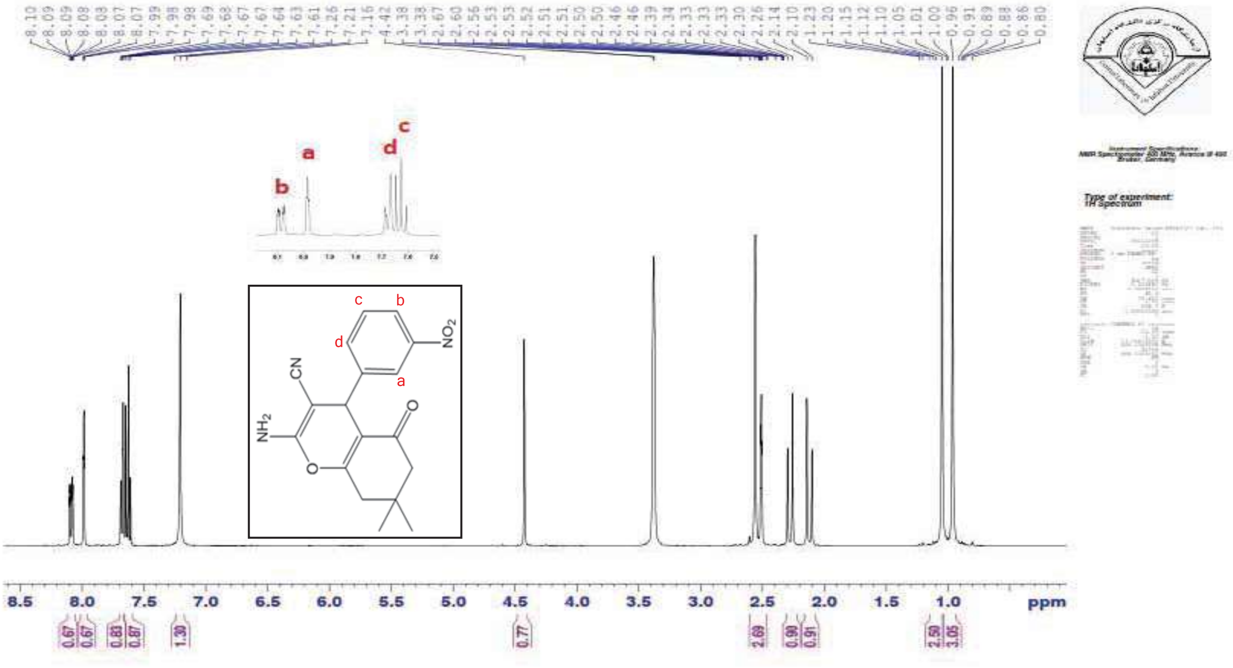

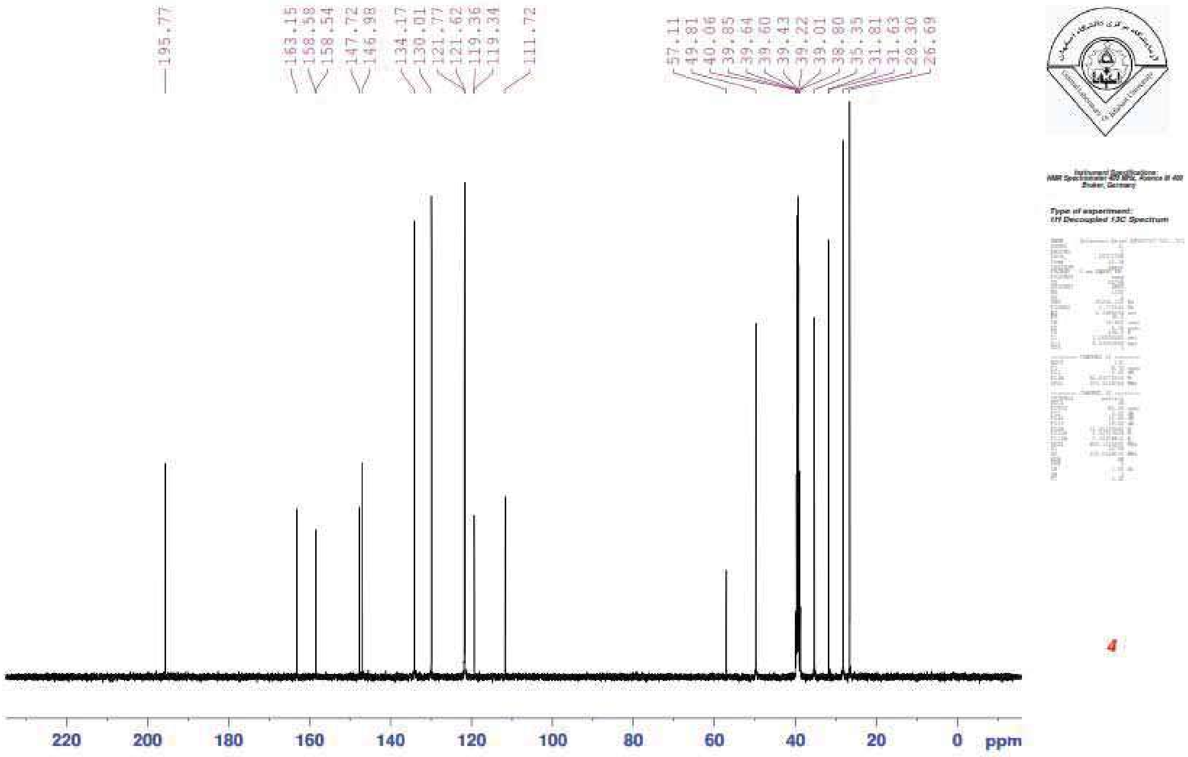

2-Amino -7, 7-dimethyl-4-(2-nitrophenyl)-5-oxo-5, 6, 7, 8-tetrahydrobenzo[*b*]pyran (Table 3, 4g). Yellow solid, mp 232–234 °C. FT-IR (ATR)/υ(cm^{-1}): 3470 (NH_2), 3334 (NH_2), 2193 (CN), 1688 (C=O), 1663 (C=C), 1525, 1364 (NO_2), 1214 (C-O); ^{1}H NMR (400MHz, DMSO-d_6) δ (ppm): 0.88 (s, 3H), 1.02 (s, 3H), 2.02 (d, 1H, J = 16.0 Hz), 2.21 (d, 1H, J = 16.0 Hz), 2.44-2.57 (m, 2H), 4.94 (s, 1H), 7.22 (s, 2H, NH_2), 7.36 (d, 1H, J = 8.0 Hz), 7.43 (t, 1H, J = 8.0 Hz), 7.66 (t, 1H, J = 8.0 Hz), 7.82 (d, 1H, J = 8.0 Hz); ^{13}C NMR (100MHz, DMSO-d_6) δ (ppm): 26.6, 28.2, 29.8, 31.8, 49.5, 56.2, 112.2, 119.0, 123.6, 127.8, 130.2, 133.3, 138.9, 148.9, 159.1, 162.6, 195.8.

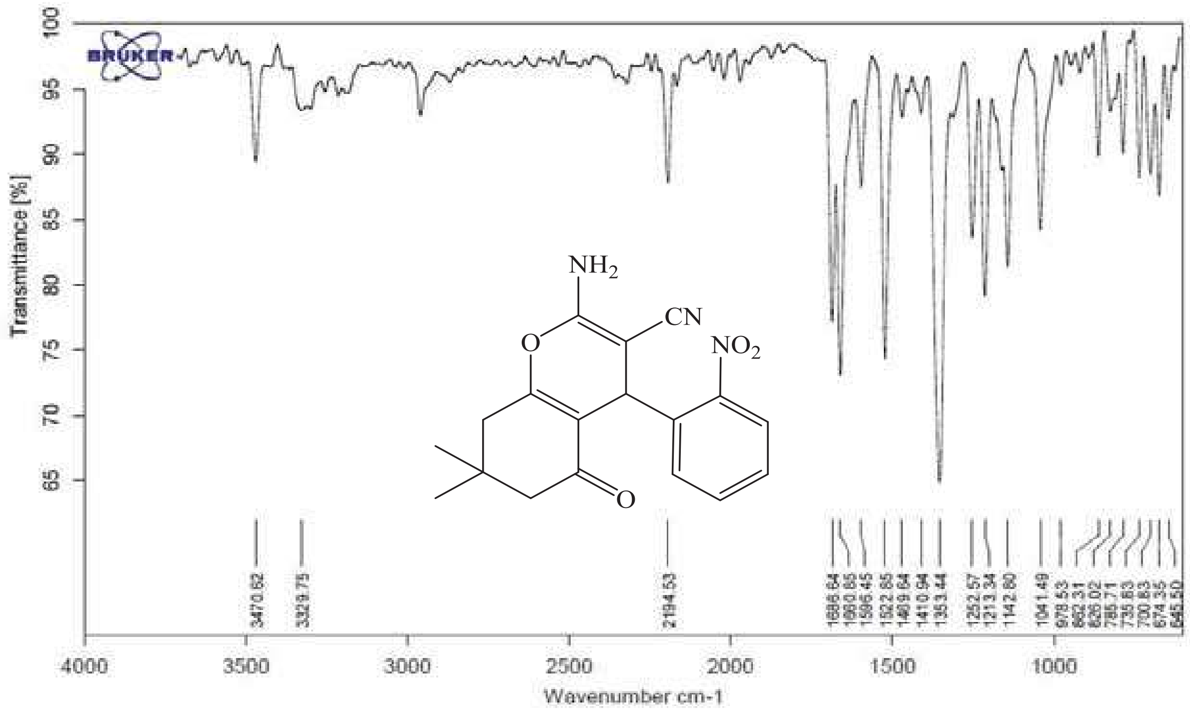

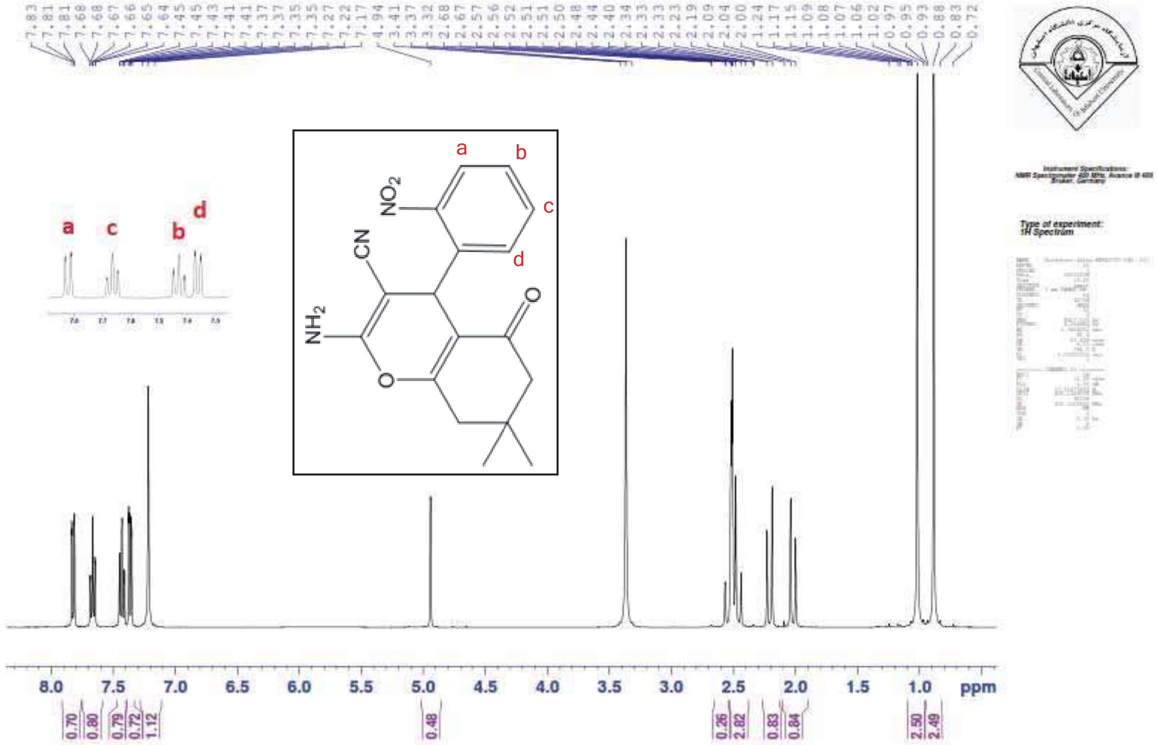

7.83 7.81 7.68 7.67 7.66 7.65 7.64 7.45 7.45 7.43 7.41 7.41 7.37 7.37 7.35 7.35 7.27 7.22 7.17 4.94 3.41 3.37 3.32 2.68 2.67 2.57 2.56 2.52 2.51 2.50 2.48 2.44 2.40 2.34 2.33 2.33 2.23 2.19 2.09 2.04 2.00 1.24 1.17 1.15 1.09 1.08 1.07 1.06 1.02 0.97 0.95 0.93 0.88 0.83 0.72
a
b
c
d
NO_2
CN
NH_2
O
Instrument Specifications: NMR Spectrometer 400 MHz, Avance III 400 Bruker, Germany
Type of experiment: 1H Spectrum
8.0 7.5 7.0 6.5 6.0 5.5 5.0 4.5 4.0 3.5 3.0 2.5 2.0 1.5 1.0 ppm
0.70 0.80 0.79 0.72 1.12 0.48 0.26 2.82 0.83 0.84 2.50 2.49

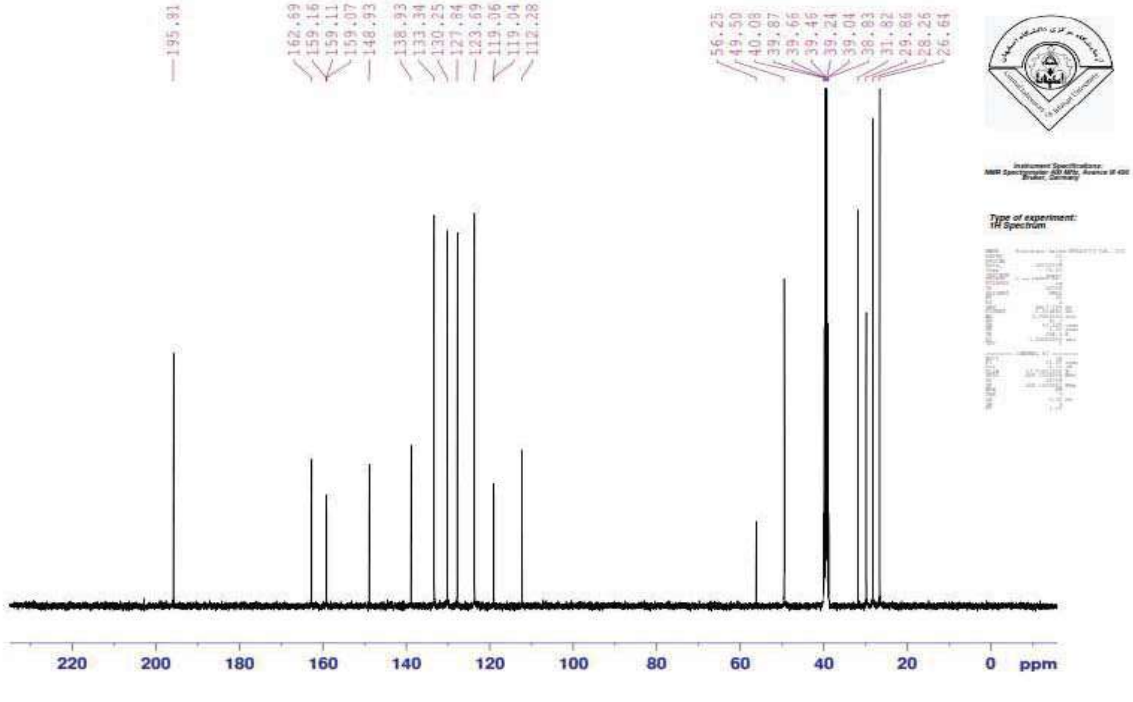

195.81
162.69 159.16 159.11 159.07 148.93 138.93 133.34 130.25 127.84 123.69 119.06 119.04 112.28
56.25 49.50 40.08 39.87 39.66 39.46 39.24 39.04 38.83 31.82 29.88 28.26 26.64
Instrument Specifications: NMR Spectrometer 400 MHz, Avance III 400 Bruker, Germany
Type of experiment: 1H Spectrum
220 200 180 160 140 120 100 80 60 40 20 0 ppm

2-Amino-3-cyano-4-(4bromophenyl)-7,7-dimethyl-5-oxo-4*H*-5,6,7,8tetrahydrobenzo[*b*]pyran (Table 3, 4h). White powder, mp 199–201 °C, FT-IR (ATR)/υ(cm^{-1}): 3387 (NH_2), 3176 (NH_2), 2190 (C≡N), 1675 (C=O), 1631(C=C), 1213(C–O).

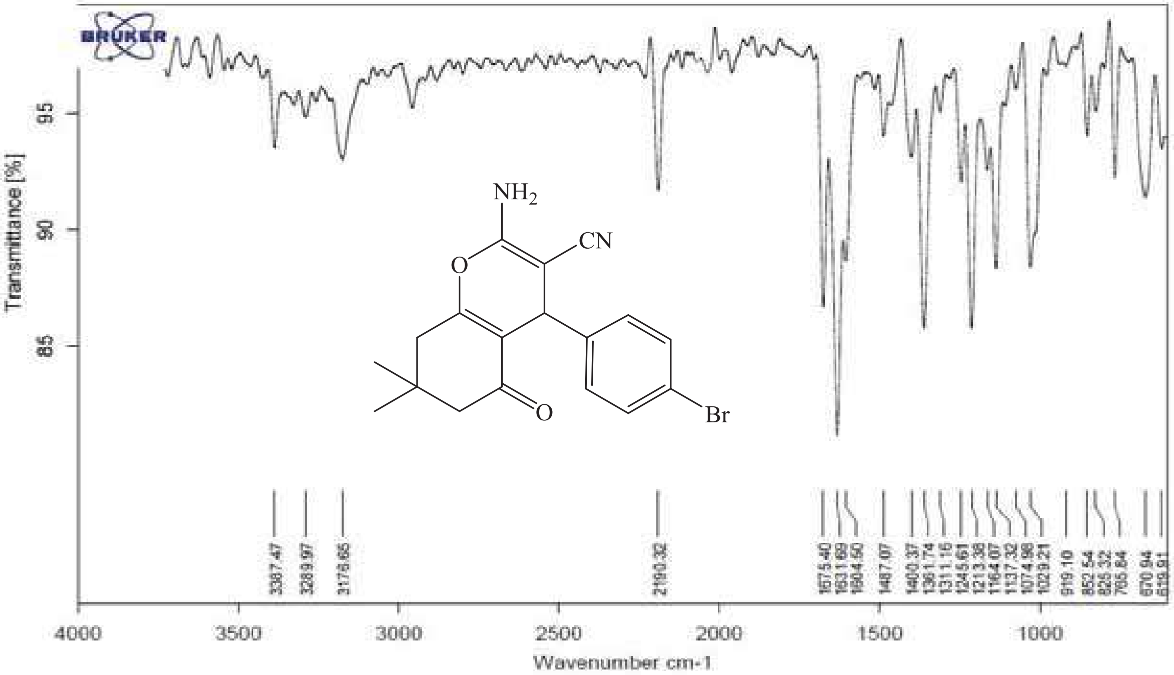

2-Amino-3-cyano-4-(4-fluorophenyl)-7,7-dimethyl-5-oxo-4*H*-5,6,7,8tetrahydrobenzo[*b*]pyran (Table 3, 4i).White powder, mp 192–194 °C, FT-IR (ATR)/υ(cm^{-1}): 3353(NH_2), 3171 (NH_2), 2189 (C≡N), 1672 (C=O), 1633(C=C), 1215(C–O). ^{1}H NMR (400 MHz, DMSO-d$_6$) δ (ppm): 0.93 (s, 3 H), 1.02 (s, 3 H), 2.11 (d, J = 16.0 Hz, 1 H), 2.25 (d, J = 16.0 Hz, 1 H), 2.45–2.54 (m, 2 H), 4.18 (s, 1 H), 7.02 (s, 2 H, NH_2), 7.09–7.16 (m, 4 H).

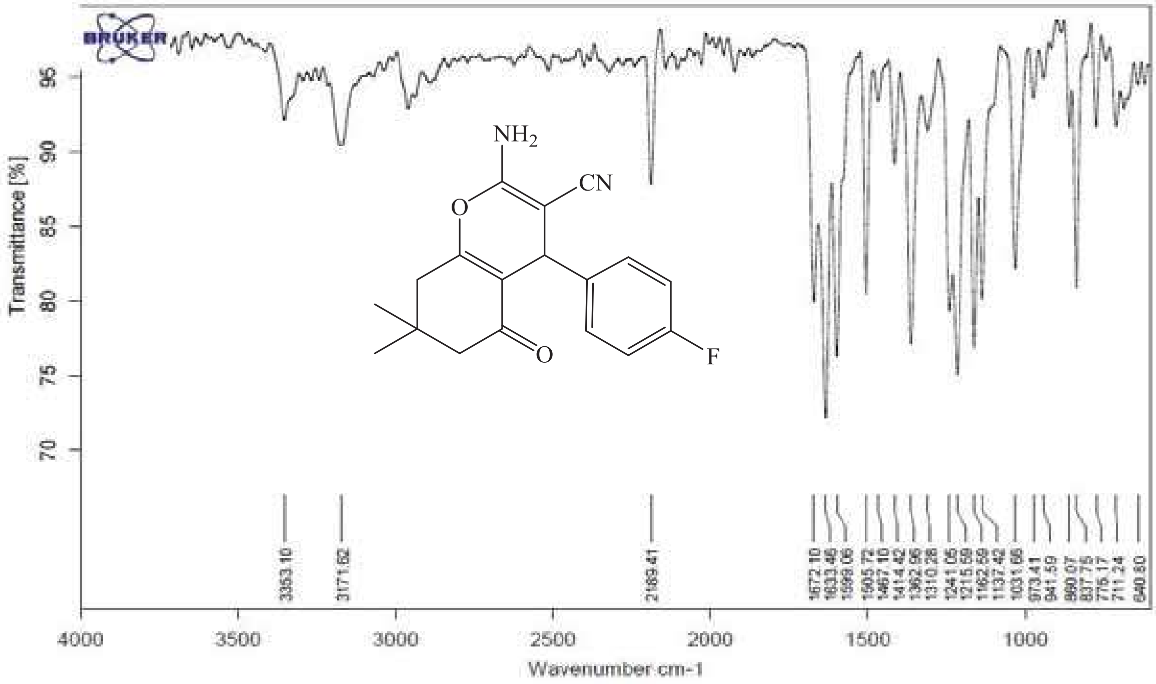

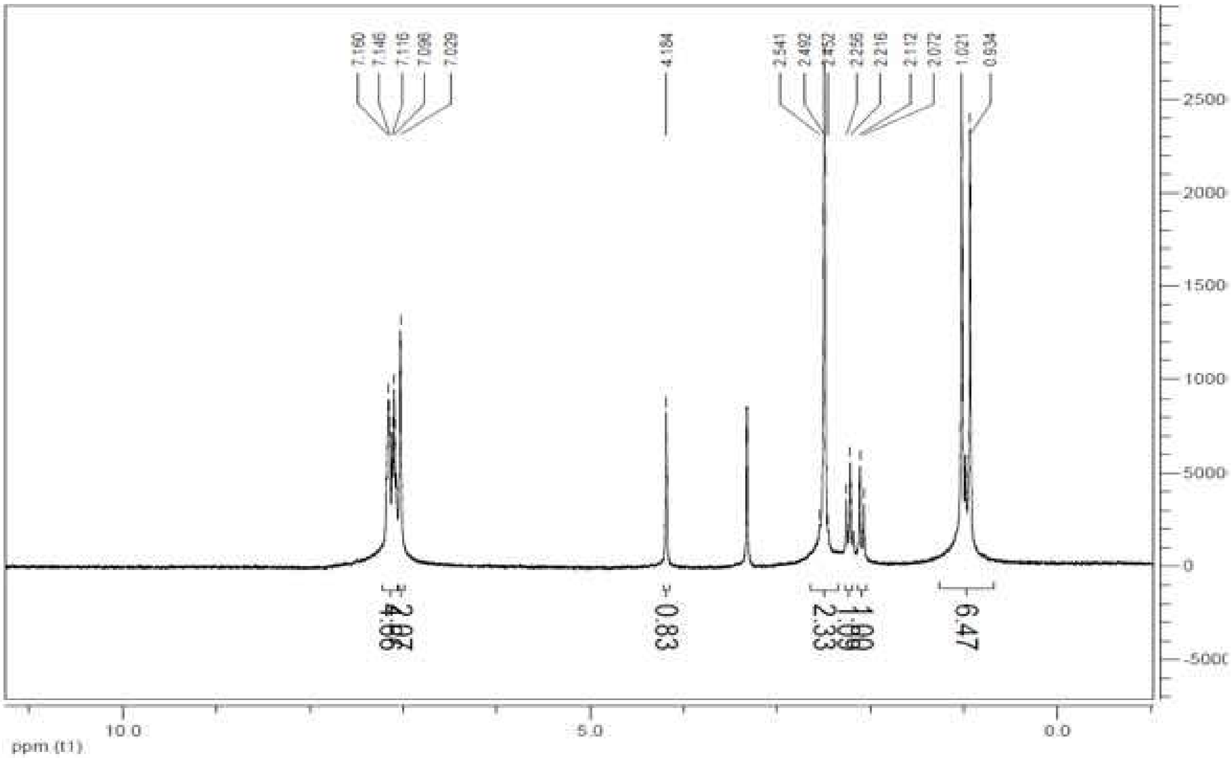

2-Amino-3-cyano-4-(4-cyanophenyl)-7,7-dimethyl-5-oxo-4*H*-5,6,7,8 tetrahydrobenzo[*b*]pyran (Table 3, 4j). White solid, mp 231–233 °C. FT-IR (ATR)/υ(cm^{-1}): 3379 (NH_2), 3351(NH_2), 2231(CN), 2193 (CN), 1684 (C=O), 1667 (C=C), 1212 (C-O).

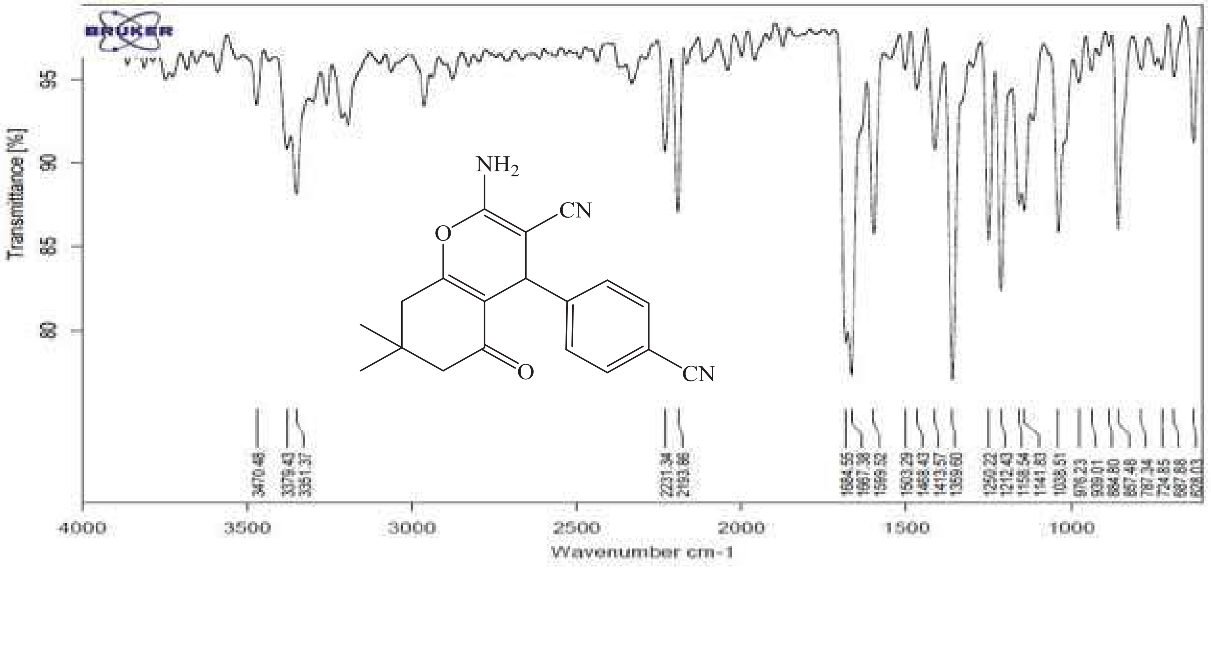

2-Amino-3-cyano-4-(4-methoxyphenyl)-7,7-dimethyl-5-oxo-4*H*-5,6,7,8-tetrahydrobenzo[*b*]pyran (Table 3, 4k). Pale yellow solid, mp: 208-210 °C. FT-IR (ATR)/υ(cm^{-1}): 3391(NH_2), 3329 (NH_2), 2191(CN), 1678 (C=O), 1654 (C=C), 1214 (C-O).

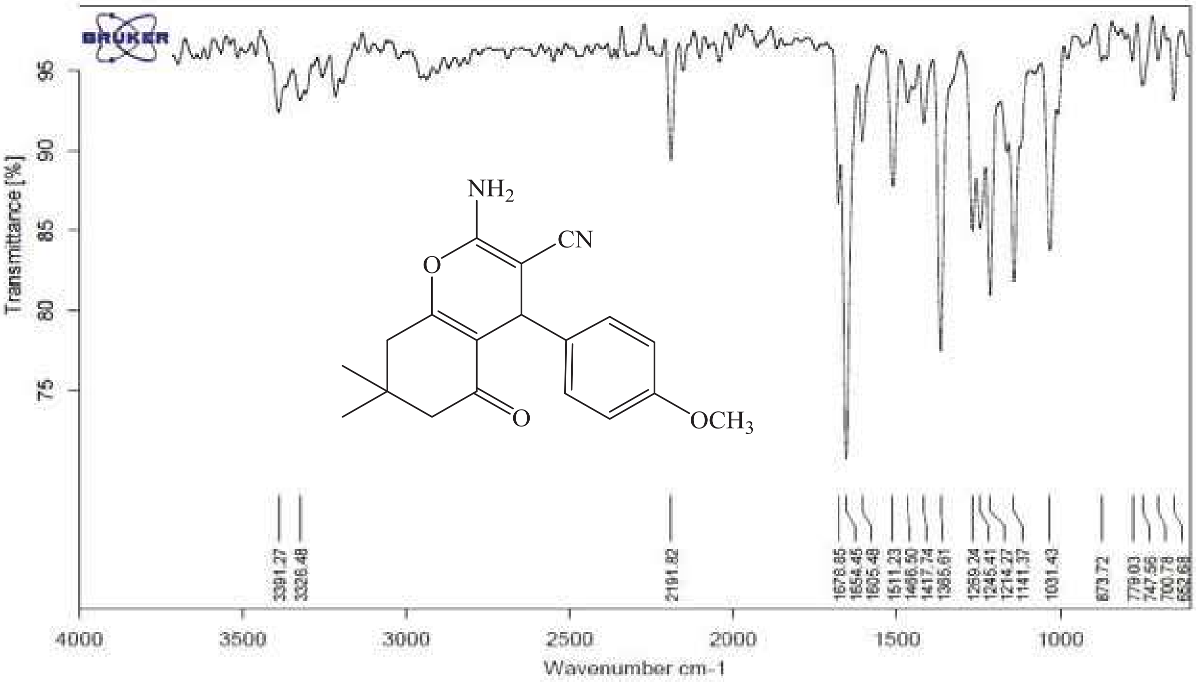

2-Amino-3-cyano-4-(3,4-dimethoxyphenyl)-7,7-dimethyl-5-oxo-4*H*-5,6,7,8-tetrahydrobenzo[*b*]pyran (Table 3, 4l). White solid, mp 175–176 °C. **FT-IR** (ATR)/υ(cm^{-1}): 3366 (NH_2), 3314 (NH_2), 2192 (CN), 1682 (C=O), 1650 (C=C), 1211 (C-O).

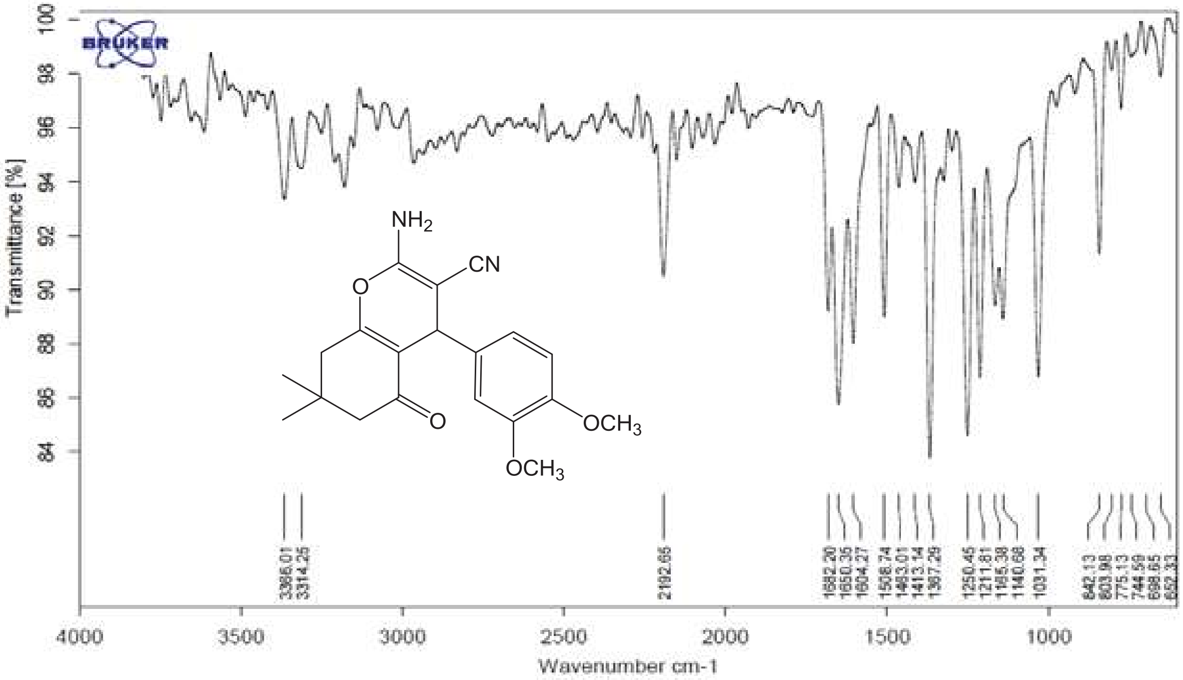

2-Amino-3-cyano-4-(4-hydroxyphenyl)-7,7-dimethyl-5-oxo-4*H*-5,6,7,8tetrahydrobenzo[*b*]pyran (Table 3, 4m). Pale yellow solid, mp 217–219 °C. FT-IR (ATR)/υ(cm^{-1}): 3308 (NH_2), 3194 (NH_2), 2201 (CN), 1680 (C=O), 1653 (C=C), 1213 (C-O); ^{1}H NMR (400 MHz, DMSO-d_6) δ (ppm): 0.93 (s, 3H), 1.01 (s, 3H), 2.09 (d, *J* = 16.0 Hz, 1H), 2.24 (d, *J* = 16.0 Hz, 1H), 2.42–2.52 (m, 2H), 4.04 (s, 1H), 6.63-6.65 (d, 2H, NH_2), 6.9-6.92 (m, 4H), 9.24 (s, 1H, OH).

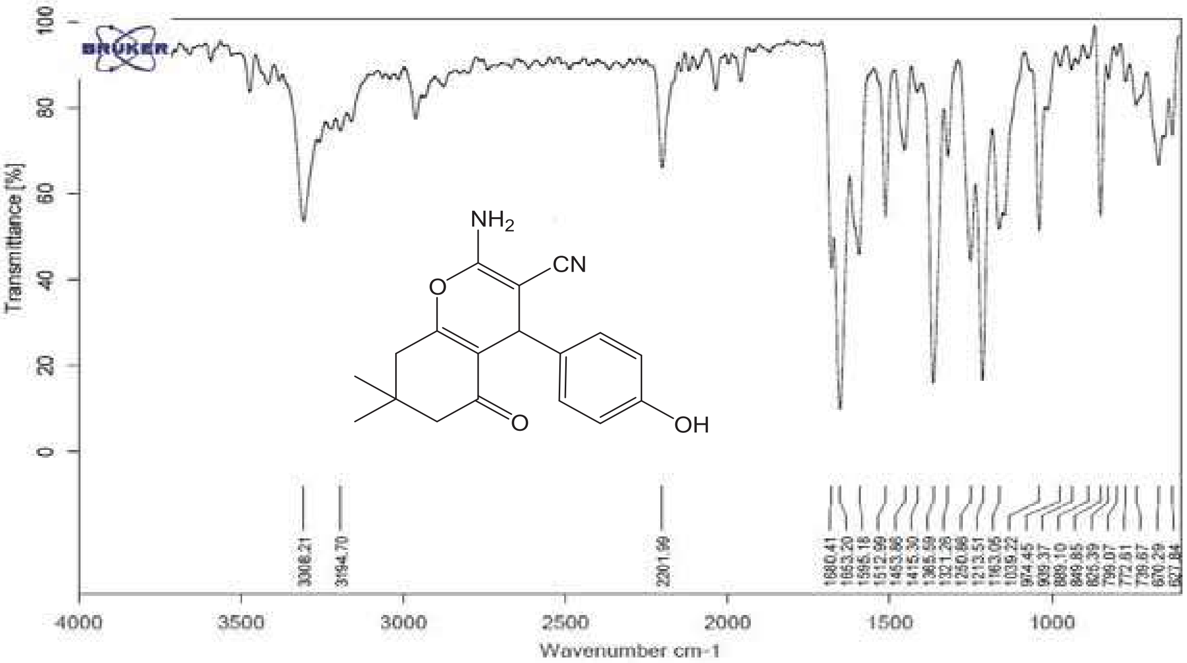

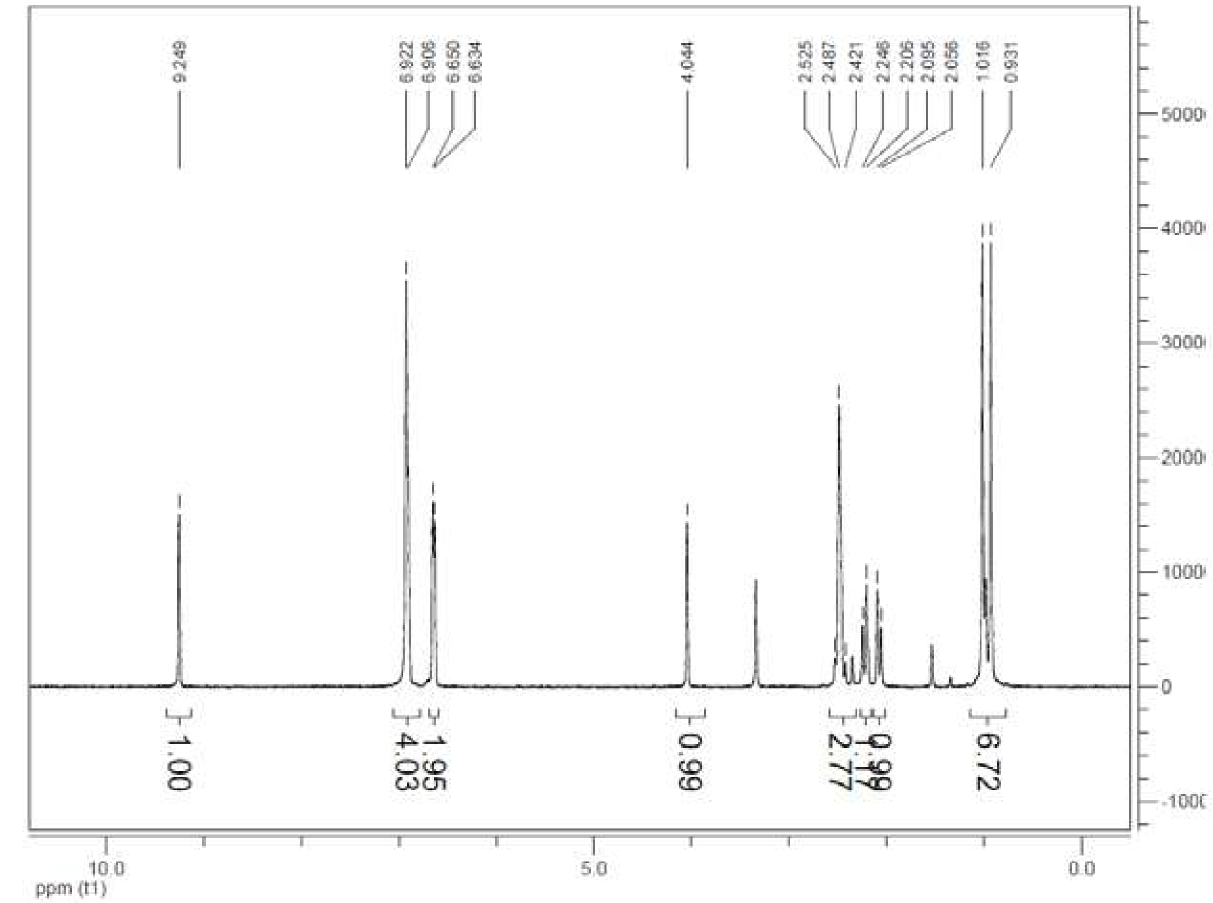

2-Amino-4-(4-isopropylphenyl)-3-cyano-7,7-dimethyl-5-oxo-4H-5,6,7,8-tetrahydrobenzo[*b*]pyran (table 3, 4n). Yellow solid, mp 197–199 °C. FT-IR (ATR)/υ(cm^{-1}): 3364 (NH_2), 3305 (NH_2), 2185 (CN), 1679 (C=O), 1645 (C=C), 1213 (C-O); ^1H NMR (400 MHz, DMSO-d_6) δ (ppm): 0.96 (s, 3H), 1.03 (s, 3H), 1.17 (d, *J* = 6.8 Hz, 6H), 1.22 (d, *J* = 6.8 Hz, 2H), 2.08–2.35 (m, 2H), 4.11 (s, 1H), 6.97 (s, 2H, NH_2), 7.04 (d, *J* = 7.6 Hz, 2H), 7.15 (d, *J* = 7.6 Hz, 2H).

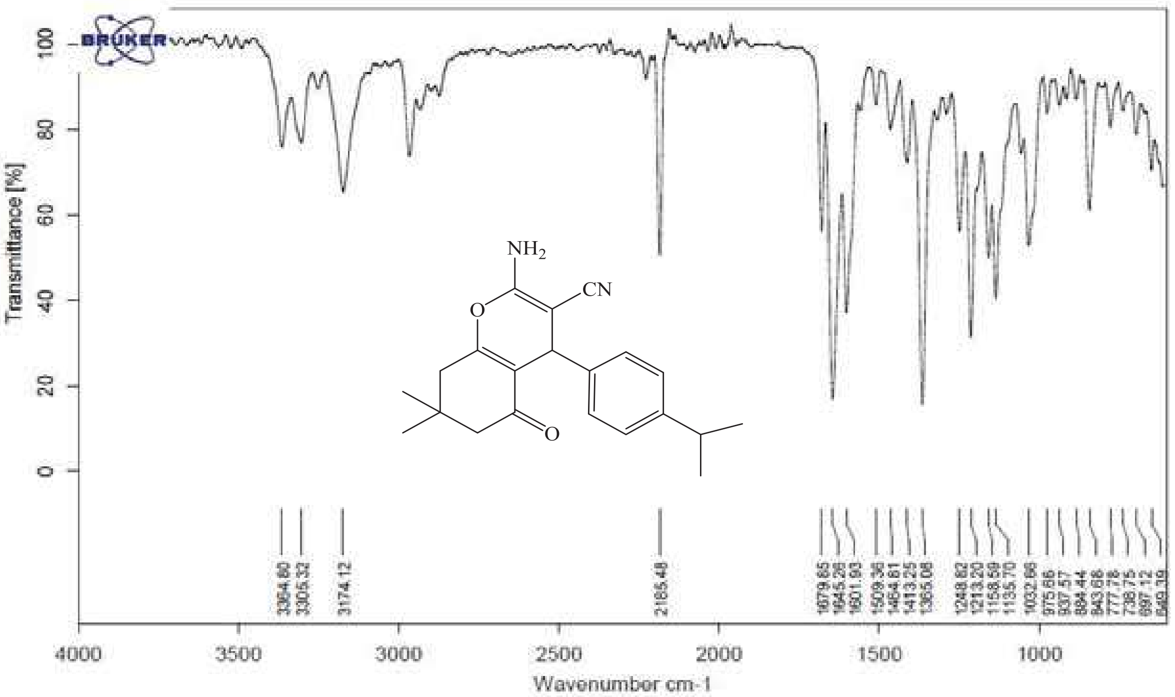

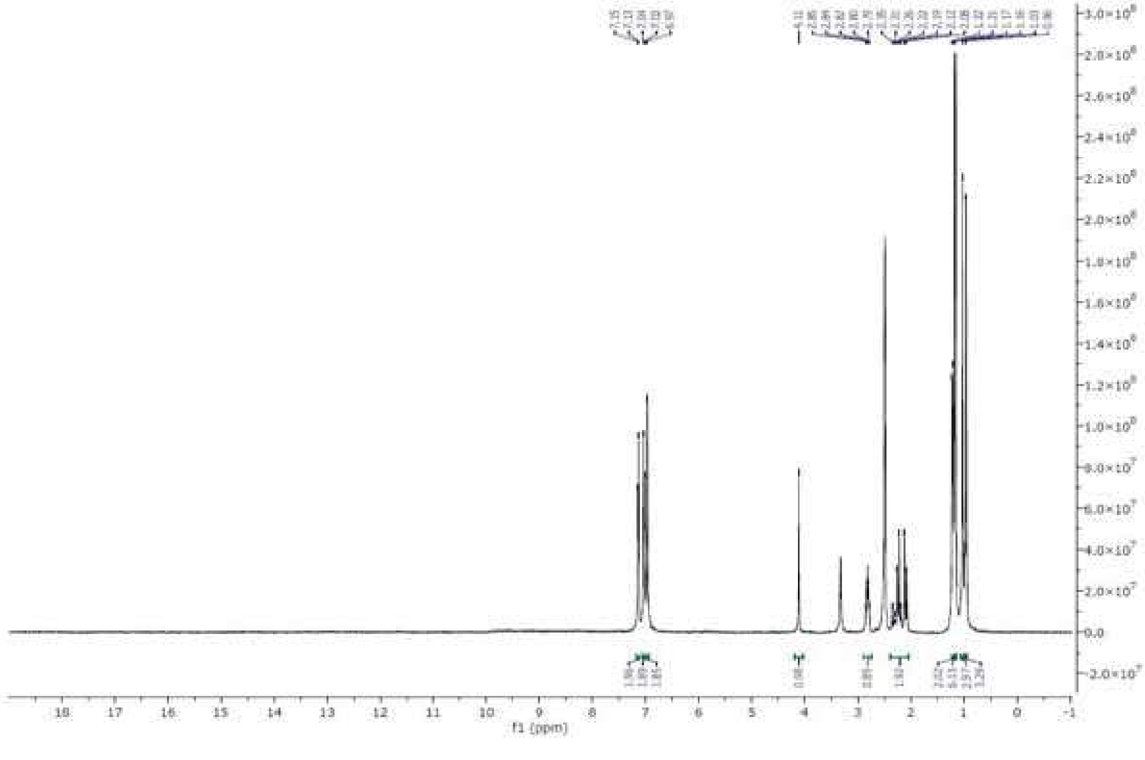

Methyl 4-(2-amino-3-cyano-7,7-dimethyl-5-oxo-4H-5,6,7,8-tetrahydrobenzo[*b*]pyran-4-yl)benzoate (Table 3, 4o). White powder, mp 257–259 °C. FT-IR (ATR)/υ(cm^{-1}): 3376 (NH_2), 3311 (NH_2), 2192 (CN), 1717 (C=O Ester), 1684 (C=O Ketone), 1646 (C=C), 1213 (C–O). ^{1}H NMR (400 MHz, DMSO-d_6) δ (ppm): 0.94 (s, 3H), 1.04 (s, 3H), 2.08 (d, *J* = 16.0 Hz, 1H), 2.24 (d, *J* = 16.0 Hz, 1H), 2.49–2.56 (m, 2H), 3.81 (s, 3H), 4.25 (s, 1H), 7.09 (s, 2H, NH_2), 7.28 (d, *J* = 8.4 Hz, 2H), 7.87(d, *J* = 8.4 Hz, 2H).^{13}C NMR (100 MHz, DMSO-d_6) δ (ppm): 26.77, 28.30, 31.79, 35.60, 40.11, 49.87, 52.02, 57.43, 112.10, 119.45, 127.63, 128.25, 129.33, 150.06, 158.51, 162.78, 165.99, 195.63.

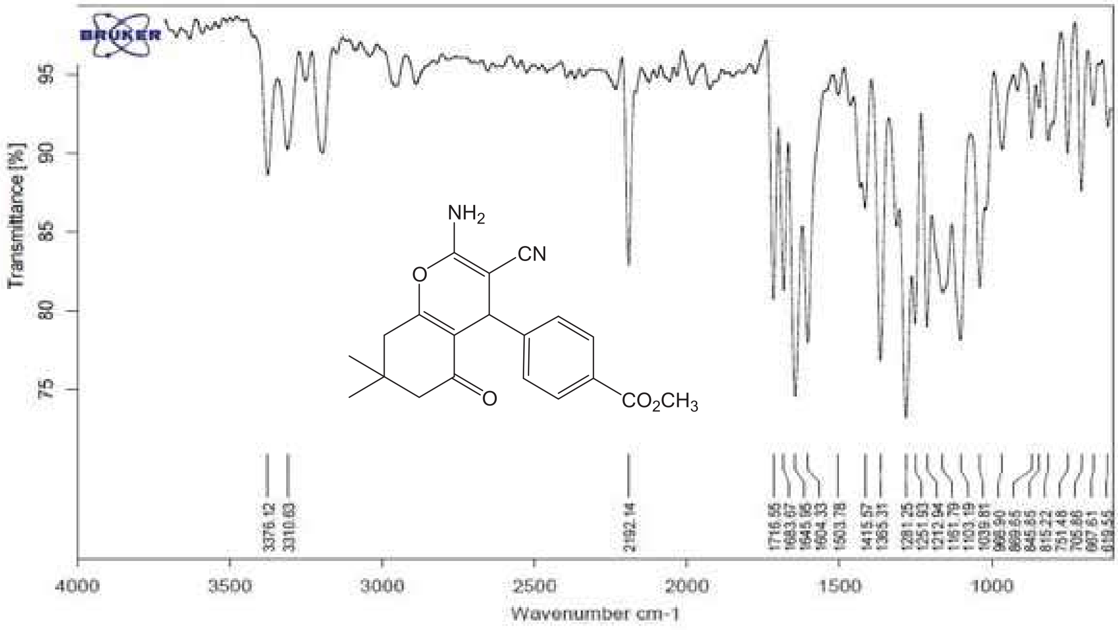

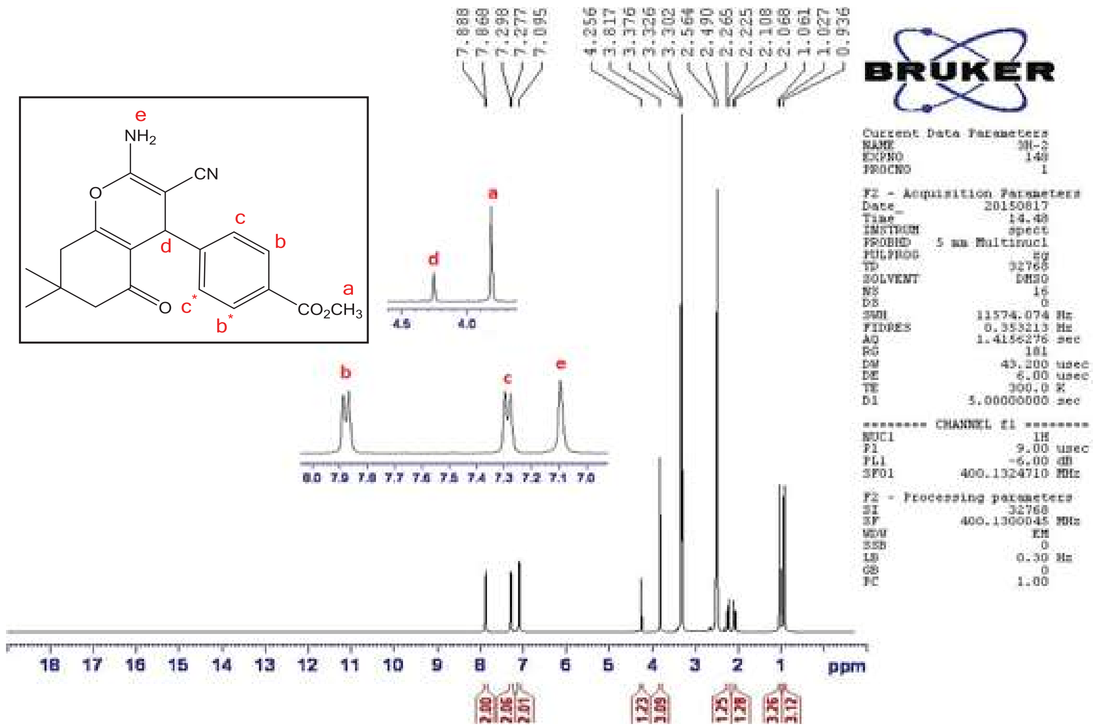

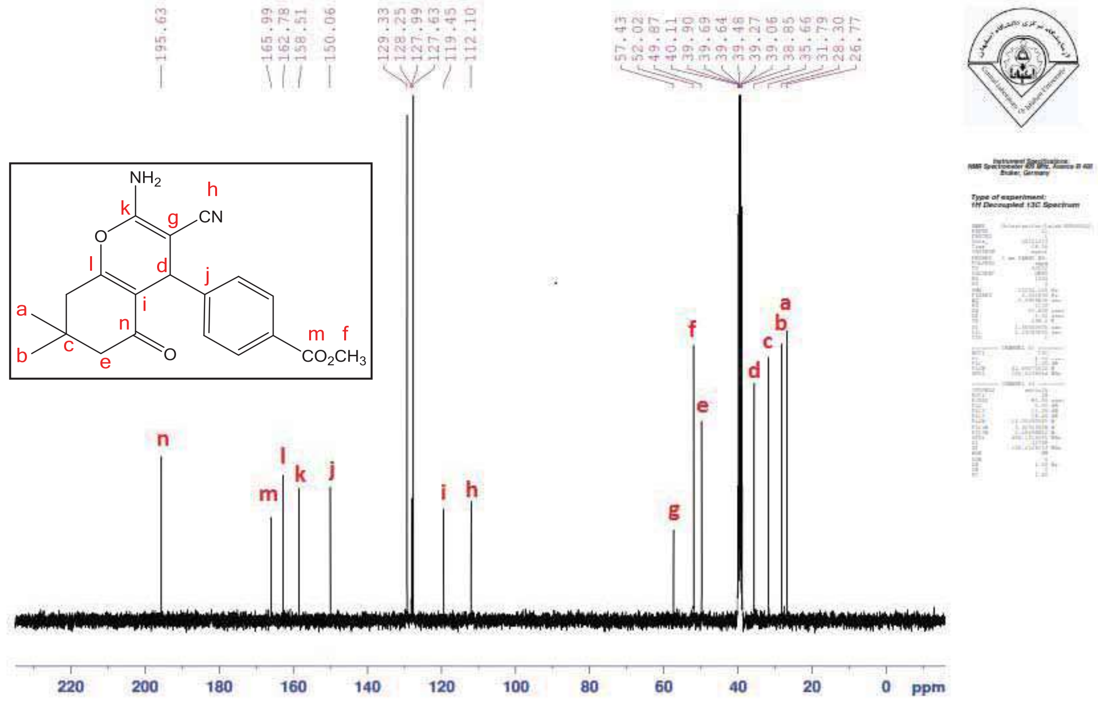

4,4´-(1,4-Phenylene)bis(2-amino-3-cyano-7,7-dimethyl-5-oxo-4H-5,6,7,8-tetrahydrobenzo-[*b*]pyran (Table 3, 4p). White powder, mp 285 (dec.) °C, FTIR (KBr, cm^{-1}): 3452 (NH_2), 3334 (NH_2), 2191 (CN), 1680 (C=O), 1664 (C=C), 1214 (C–O). ^{1}H NMR (400 MHz, DMSO-d_6) δ (ppm): 1.04 (s, 6 H), 1.10 (s, 6 H), 2.17–2.38 (m, 4 H), 2.52–2.62 (m, 4 H), 4.20 (s 2 H), 7.02 (s, 4 H, $2NH_2$), 7.10 (s, 4H, Ar-H). ^{13}C NMR (100 MHz, DMSO-d_6), δ (ppm): 26.93, 27.19, 28.22, 31.84, 34.96, 49.93, 58.35, 112.82, 119.77, 126.94, 142.85, 158.55, 162.67, 195.66.

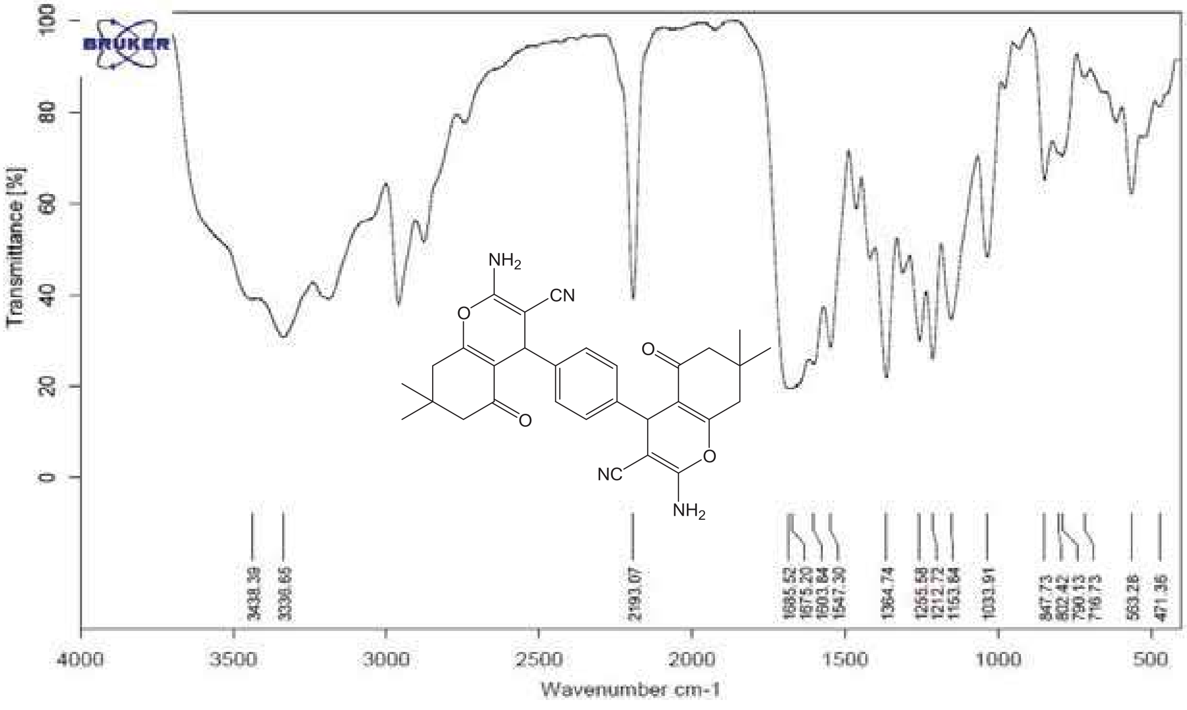

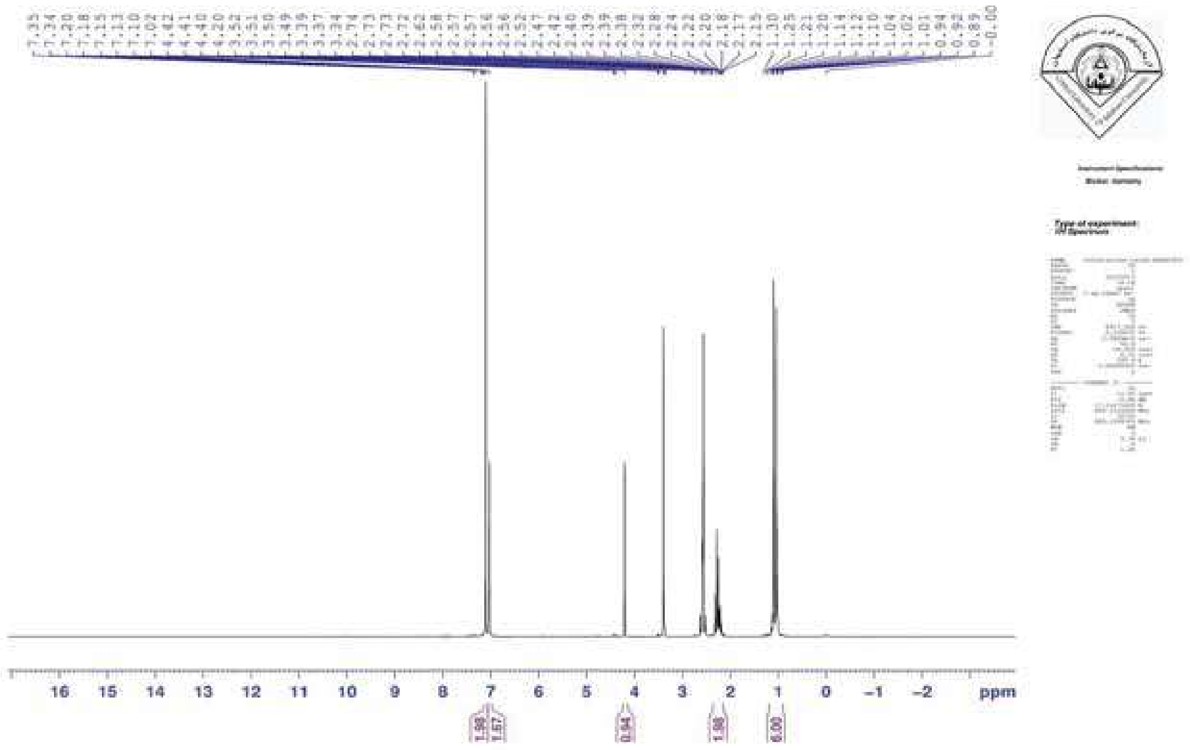

Type of experiment:
1H Spectrum
16 15 14 13 12 11 10 9 8 7 6 5 4 3 2 1 0 −1 −2 ppm
1.98
1.97
0.94
1.98
6.00

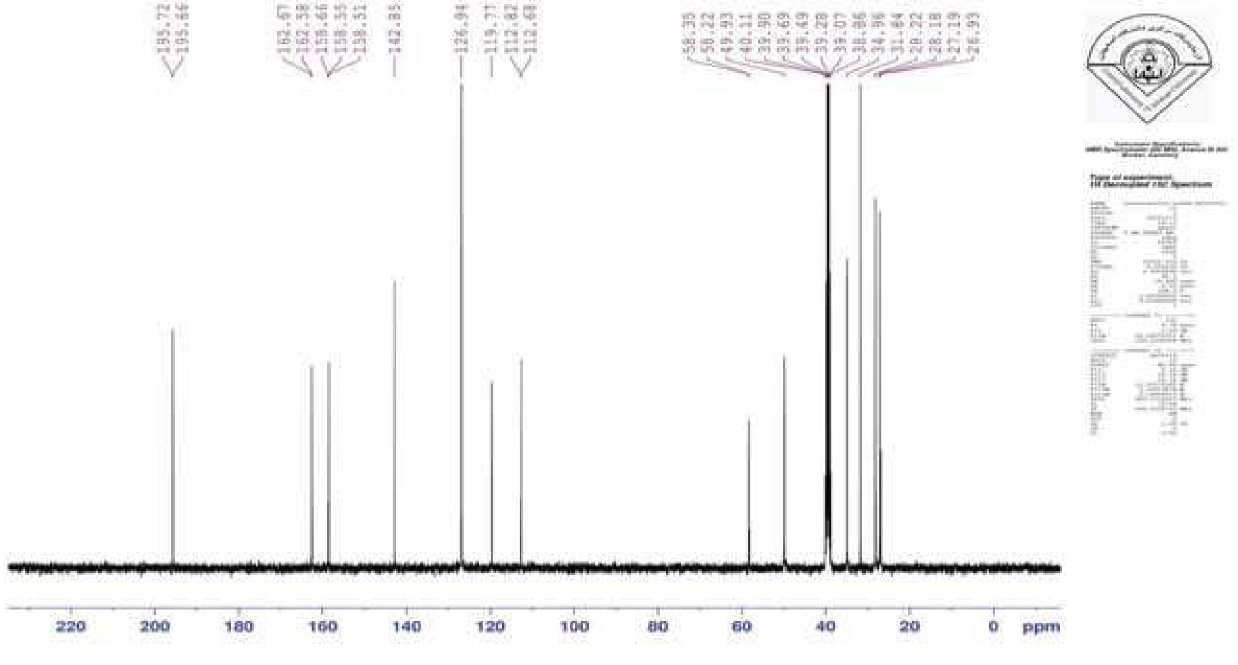

195.72
195.66
162.67
162.38
158.66
158.55
158.51
142.85
126.94
119.77
112.82
112.68
58.35
50.22
49.93
40.11
39.90
39.69
39.49
39.28
39.07
38.86
34.96
31.84
28.22
28.18
27.19
26.93
Type of experiment:
1H Decoupled 13C Spectrum
220 200 180 160 140 120 100 80 60 40 20 0 ppm

2-Amino-3-cyano-4-(furan-2-yl)-7,7-dimethyl-5-oxo-4H-5,6,7,8-tetrahydrobenzo[*b*]pyran (Table 3, 4q). Cream powder, mp 216–218 °C. FT-IR (ATR)/υ(cm^{-1}): 3390 (NH_2), 3327 (NH_2), 2194 (CN), 1677 (C=O), 1665 (C=C), 1215 (C–O). ^{1}H NMR (400 MHz, DMSO-d_6) δ (ppm): 0.99 (s, 3 H), 1.05 (s, 3H), 2.15 (d, *J* = 16.0 Hz, 1H), 2.29 (d, *J* = 16.0 Hz, 1H), 2.43–2.68 (m, 2H), 4.33 (s, 1H), 6.09 (d, *J* = 4.0 Hz, 1H), 6.31 (m, 1H), 7.12 (s, 2H, NH_2), 7.47 (d, *J* = 4.0 Hz,1H). ^{13}C NMR (100 MHz, DMSO-d_6) δ (ppm): 26.51, 28.39, 28.93, 31.79, 49.85, 55.31, 105.03, 110.31, 110.42, 119.52, 141.73, 155.67, 159.23, 163.25, 195.42.

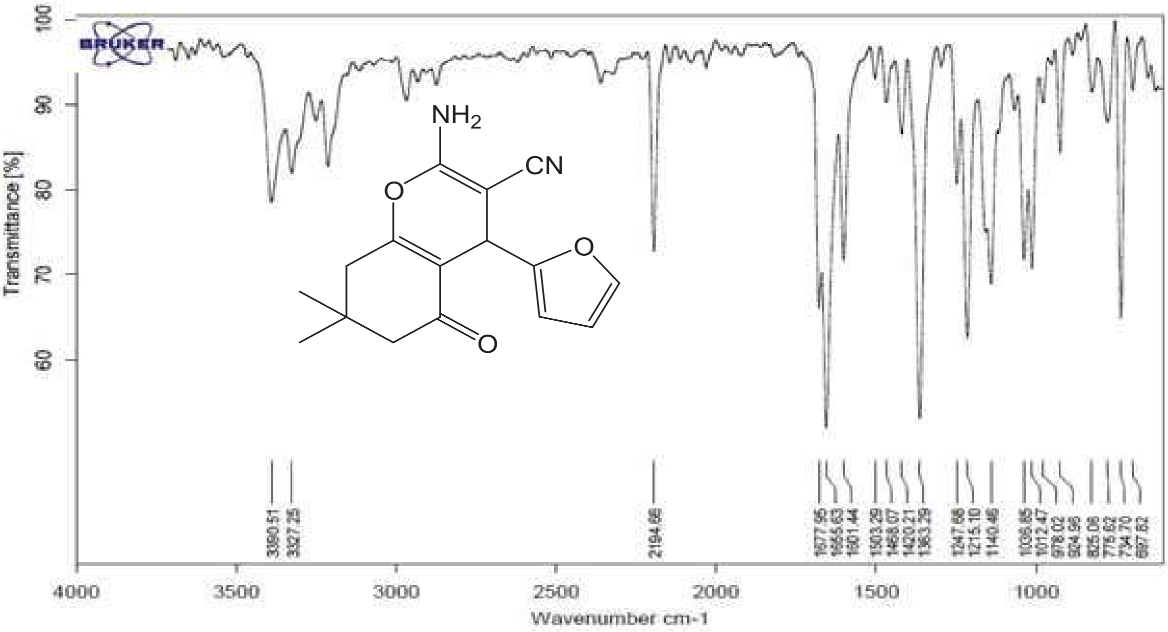

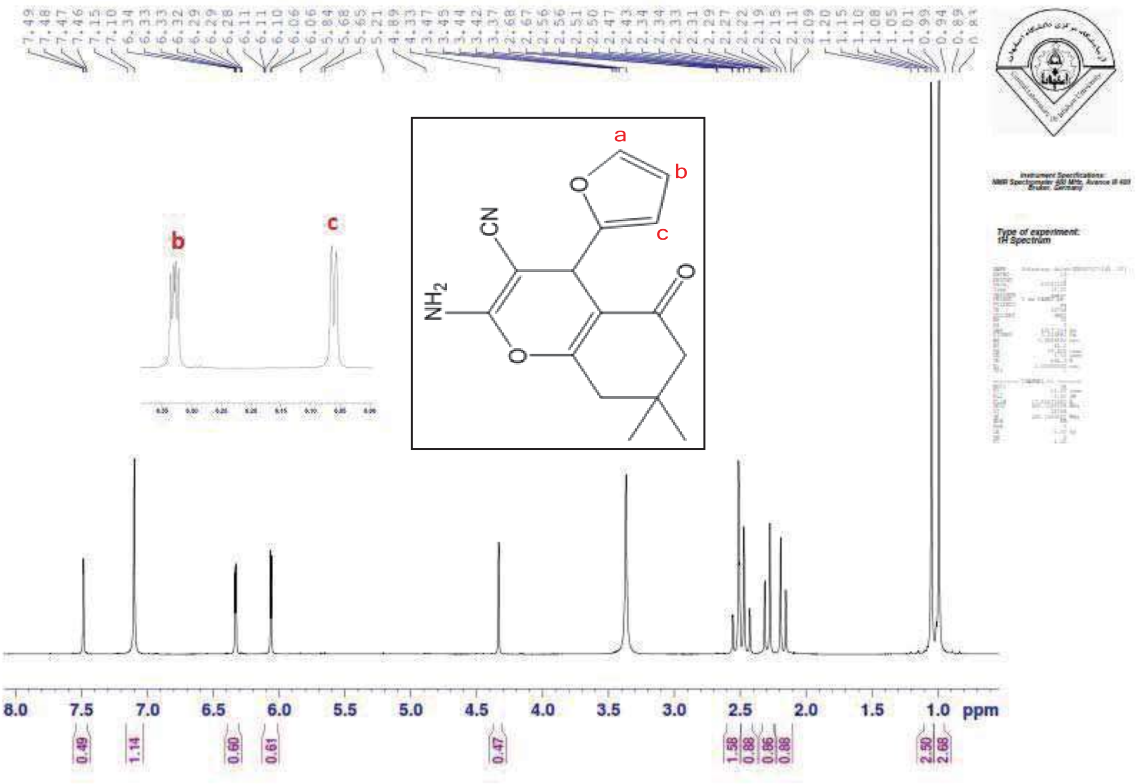

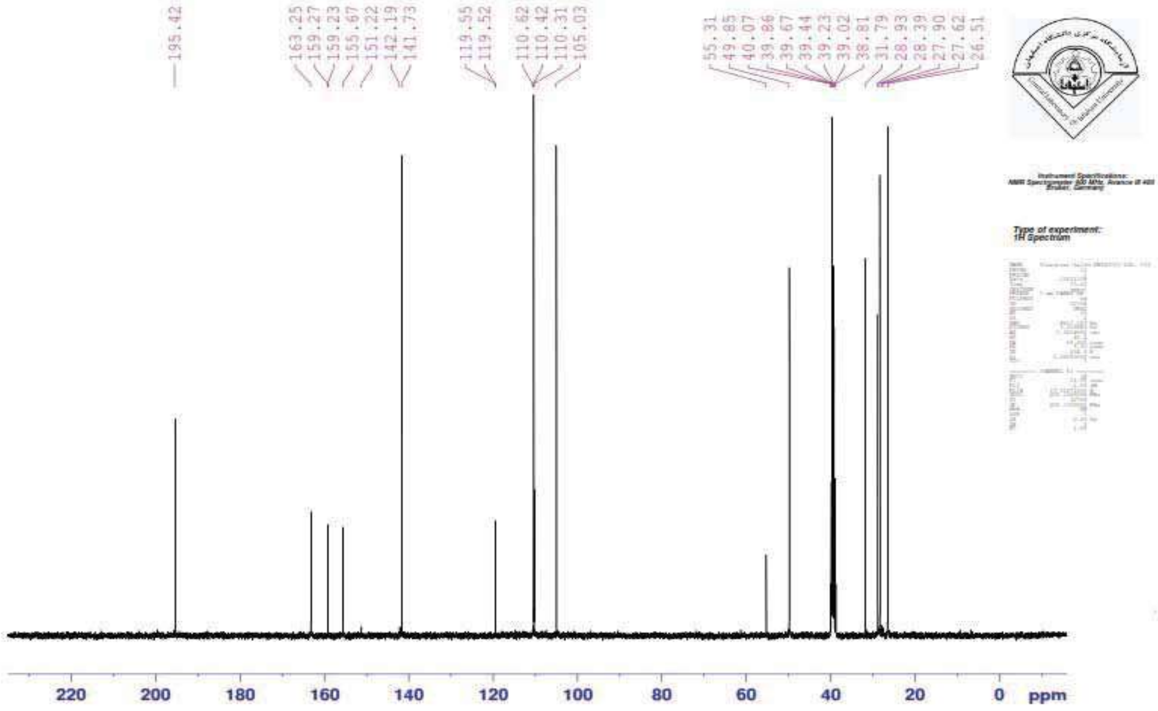

2-Amino-3-cyano-4-phenyl-7,7-dimethyl-5-oxo-4*H*-5,6,7,8-tetrahydrobenzo[*b*]pyran (Table 3, 4r). Pale yellow solid, mp 162–164 °C. FT-IR (ATR)/υ(cm^{-1}): 3387 (NH_2), 3307 (NH_2), 2186 (CN), 1680 (C=O), 1658 (C=C), 1218 (C-O); ^{1}H NMR (400MHz, $CDCl_3$) δ (ppm): 0.79 (t, 3H, CH_3, *J*=8.0 Hz), 1.02-1.04 (2s, 6H, 2CH_3), 1.11-1.30 (m, 6H, 3CH_2), 1.40-1.57 (m, 2H, CH_2), 2.17-2.37 (m, 4H, 2CH_2), 3.33 (t, 1H, *J*=8.0 Hz), 4.62 (s, 2H, NH_2); ^{13}C NMR (100MHz, $CDCl_3$) δ (ppm): 14.0, 22.5, 24.5, 27.4, 29.1, 29.3, 31.6, 32.0, 34.6, 40.6, 50.8, 60.7, 113.9, 119.4, 159.0, 162.7, 196.7.

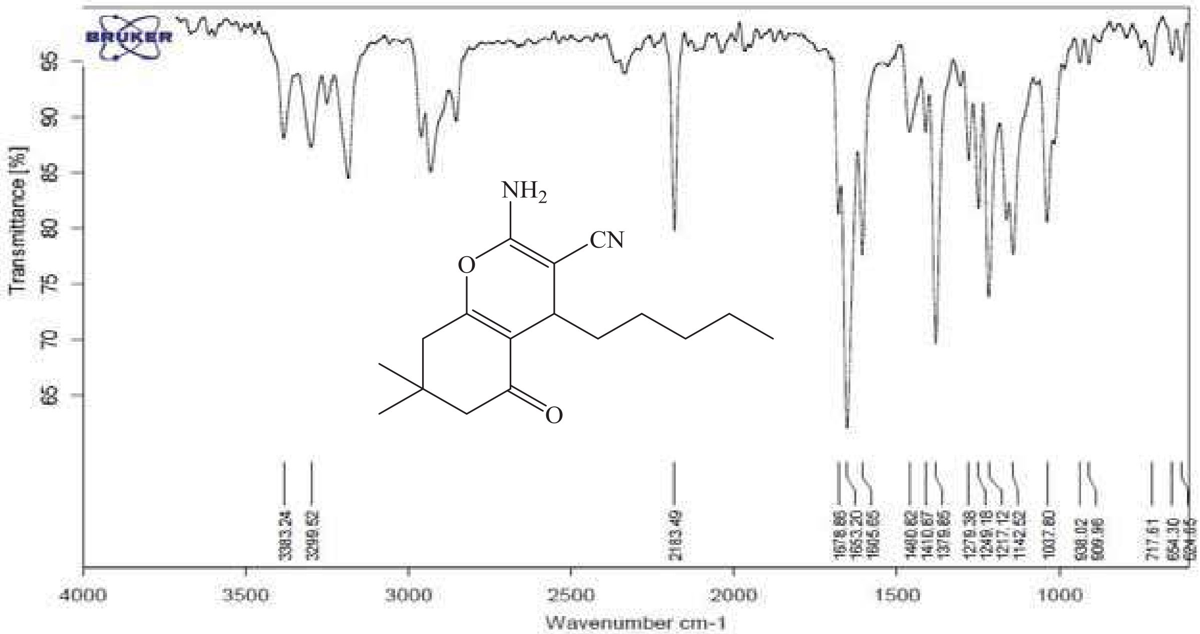

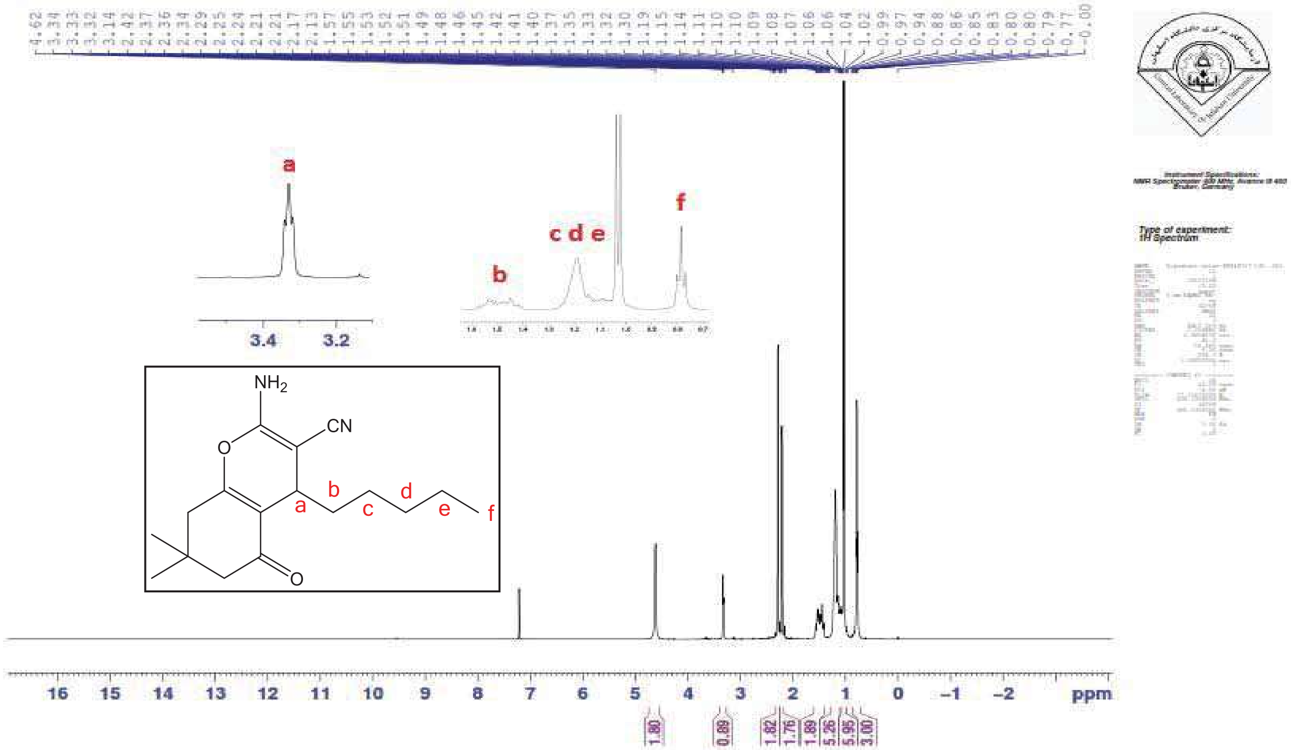
4.62 3.34 3.33 3.32 3.14 2.42 2.37 2.36 2.34 2.29 2.25 2.24 2.21 2.21 2.17 2.13 1.57 1.55 1.53 1.52 1.51 1.49 1.48 1.46 1.45 1.42 1.41 1.40 1.37 1.35 1.33 1.32 1.30 1.19 1.15 1.14 1.11 1.10 1.10 1.09 1.08 1.07 1.06 1.06 1.04 1.02 0.99 0.97 0.94 0.88 0.86 0.85 0.83 0.80 0.80 0.79 0.77 -0.00
a
3.4
3.2
b
c d e
f
NH2
CN
O
a
b
c
d
e
f
O
Instrument Specifications:
NMR Spectrometer 400 MHz, Avance III 400
Bruker, Germany
Type of experiment:
1H Spectrum
16 15 14 13 12 11 10 9 8 7 6 5 4 3 2 1 0 -1 -2 ppm
1.80
0.89
1.82
1.76
1.89
5.26
5.95
3.00

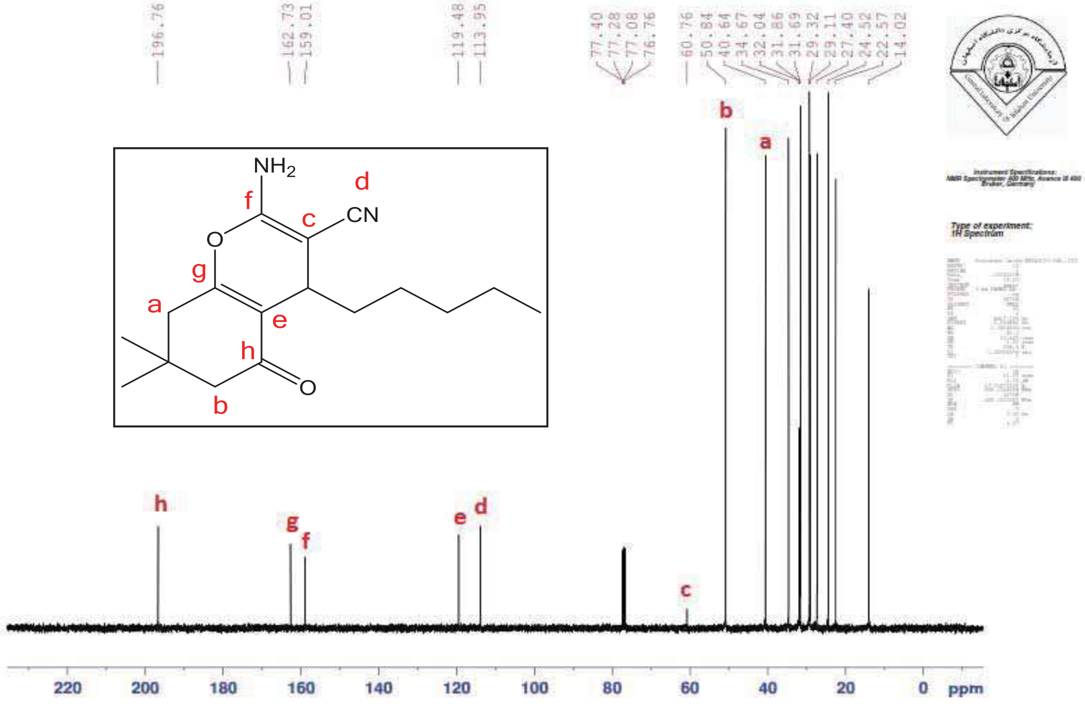
196.76
162.73
159.01
119.48
113.95
77.40
77.28
77.08
76.76
60.76
50.84
40.64
34.67
32.04
31.86
31.69
29.32
29.11
27.40
24.52
22.57
14.02
b
a
NH2
d
f
CN
c
O
g
a
e
h
O
b
Instrument Specifications:
NMR Spectrometer 400 MHz, Avance III 400
Bruker, Germany
Type of experiment:
1H Spectrum
h
g
f
e
d
c
220 200 180 160 140 120 100 80 60 40 20 0 ppm

(E)-2-amino-3-cyano-7,7-dimethyl-5-oxo-4-styryl-4H-5,6,7,8-tetrahydrobenzo[b]pyran (Table 3, 4s). Pale yellow solid, mp 217–219°C. FT-IR (ATR)/υ(cm^{-1}): 3383 (NH_2), 3292 (NH_2), 2181 (CN), 1680 (C=O), 1649 (C=C), 1215 (C–O). ^{1}H NMR (400MHz, DMSO-d_6) δ (ppm): 1.01 (s, 3H), 1.04 (s, 3H), 2.21 (d, 1H, J = 16.0 Hz), 2.29 (d, 1H, J = 16.0 Hz), 2.39-2.50 (m, 2H, CH_2), 3.82 (d, 1H, J=8.0 Hz), 6.08(dd, 1H, J=16.0, 8.0 Hz), 6.37 (d, 1H, J=16.0 Hz), 7.08(s, 2H, NH_2), 7.23(t, 1H, J = 8.0 Hz), 7.31 (t, 2H, J = 8.0 Hz), 7.39 (d, 2H, J = 8.0 Hz); ^{13}C NMR (100MHz, DMSO-d_6) δ (ppm): 26.8, 28.1, 31.8, 32.7, 50.0, 55.1, 111.8, 119.8, 126.1, 127.4, 128.3, 129.2, 131.0, 136.4, 159.1, 162.3, 195.9.

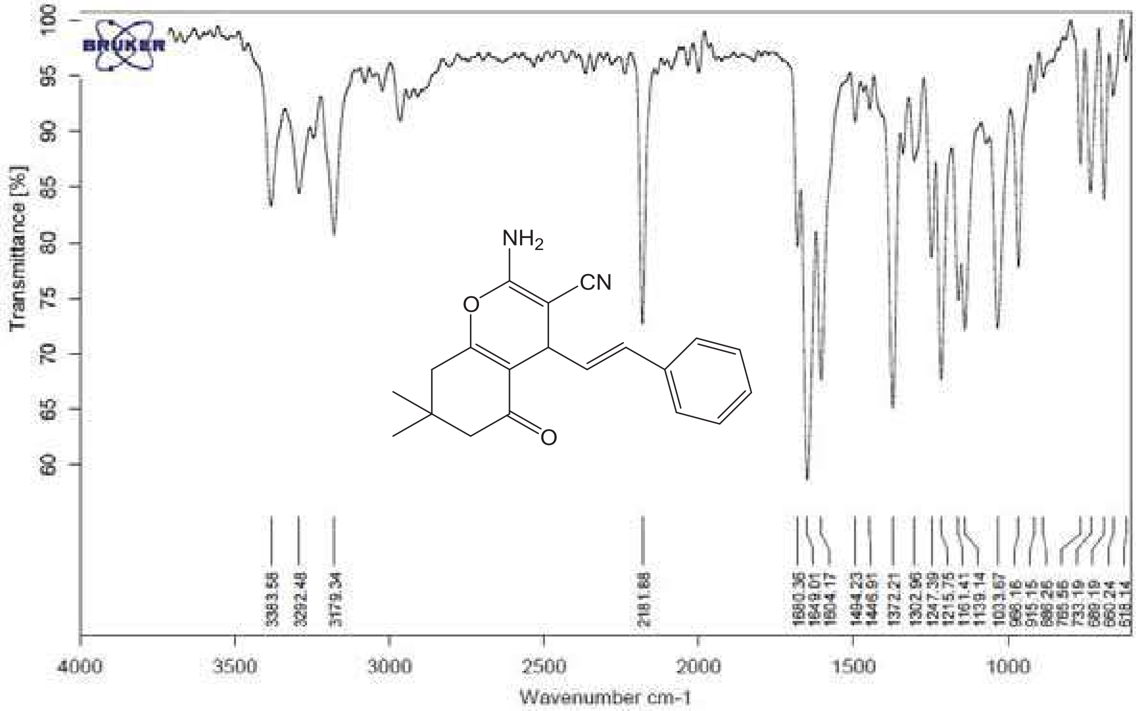

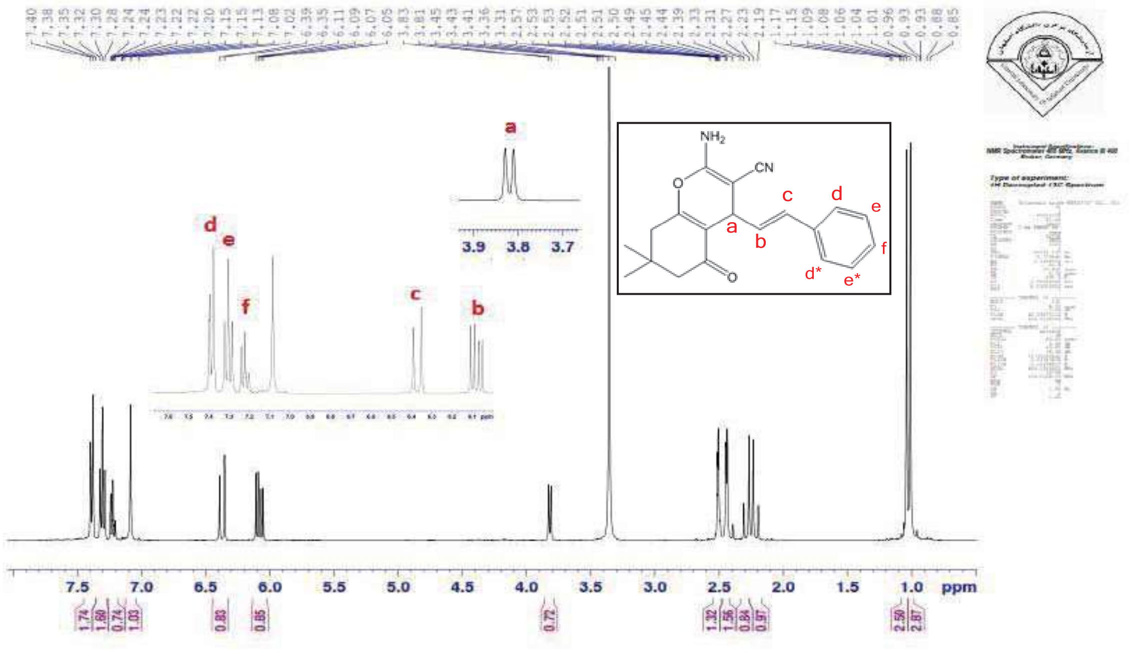

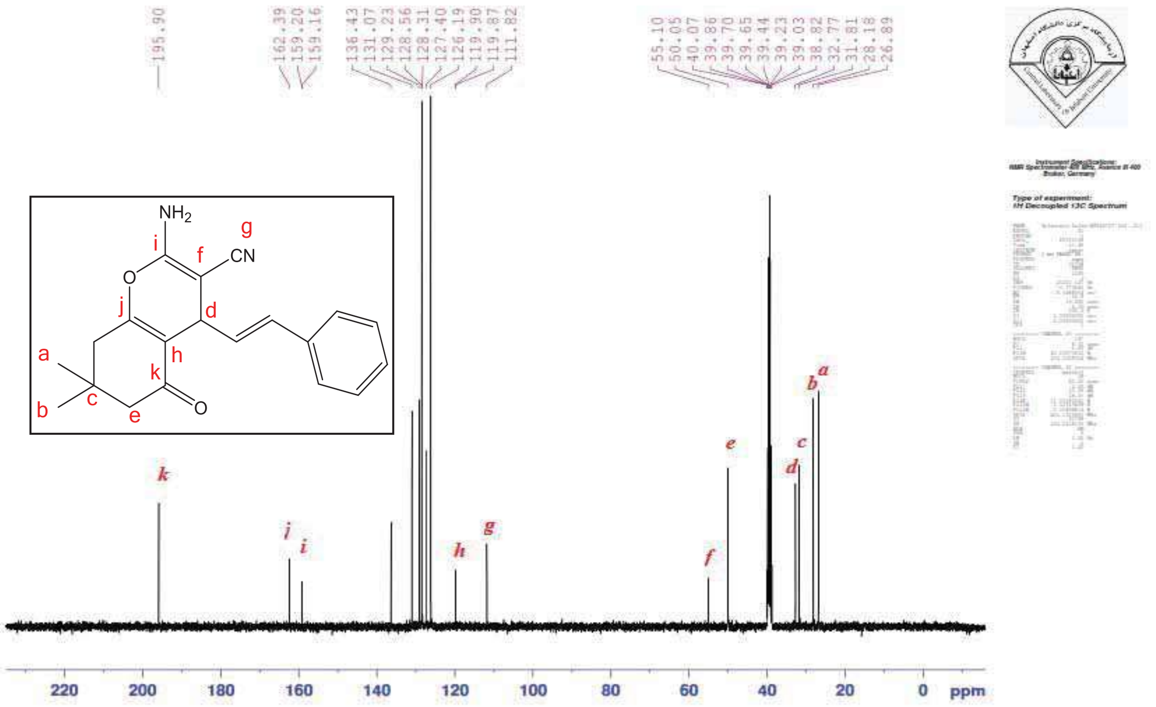
195.90
162.39
159.20
159.16
136.43
131.07
129.23
128.56
128.31
127.40
126.19
119.90
119.87
111.82
55.10
50.05
40.07
39.86
39.70
39.65
39.44
39.23
39.03
38.82
32.77
31.81
28.18
26.89
NH2
CN
O
a b c d e f g h i j k
220 200 180 160 140 120 100 80 60 40 20 0 ppm
Instrument Specifications:
NMR Spectrometer 400 MHz, Avance III 400
Bruker, Germany
Type of experiment:
1H Decoupled 13C Spectrum
